# Blocking Aerobic Glycolysis by Targeting Pyruvate Dehydrogenase Kinase in Combination with EGFR TKI and Ionizing Radiation Increases Therapeutic Effect in Non-Small Cell Lung Cancer Cells

**DOI:** 10.3390/cancers13050941

**Published:** 2021-02-24

**Authors:** Sissel E. Dyrstad, Maria L. Lotsberg, Tuan Zea Tan, Ina K. N. Pettersen, Silje Hjellbrekke, Deusdedit Tusubira, Agnete S. T. Engelsen, Thomas Daubon, Arnaud Mourier, Jean Paul Thiery, Olav Dahl, James B. Lorens, Karl Johan Tronstad, Gro V. Røsland

**Affiliations:** 1 Department of Biomedicine, University of Bergen, 5009 Bergen, Norway; dyrstadsissel@gmail.com (S.E.D.); Maria.lie@uib.no (M.L.L.); ina.pettersen@uib.no (I.K.N.P.); silje_johj@hotmail.com (S.H.); deusdedit.tusubira@gmail.com (D.T.); Agnete.Engelsen@uib.no (A.S.T.E.); james.lorens@uib.no (J.B.L.); Karl.Tronstad@uib.no (K.J.T.); 2 Centre for Cancer Biomarkers CCBIO, University of Bergen, 5009 Bergen, Norway; tjp@nus.edu.sg; 3 Department of Pathology, Haukeland University Hospital, 5021 Bergen, Norway; 4 Cancer Science Institute of Singapore, National University of Singapore, Singapore 117599, Singapore; csittz@nus.edu.sg; 5 INSERM UMR 1186, Gustave Roussy, Université Paris-Saclay, 94805 Villejuif, France; 6 University of Bordeaux, CNRS, IBGC, UMR 5095, 33000 Bordeaux, France; thomas.daubon@u-bordeaux.fr (T.D.); Arnaud.mourier@ibgc.cnrs.fr (A.M.); 7 Guangzhou Regenerative Medicine and Health, Guangdong Laboratory, Guangzhou 510530, China; 8 Department of Oncology and Medical Physics, Haukeland University Hospital, 5021 Bergen, Norway; Olav.Dahl@uib.no

**Keywords:** Warburg effect, glycolysis, PDHK, PDH, DCA, mitochondria, NSCLC, ionizing radiation, EGFR TKI

## Abstract

**Simple Summary:**

Non-small cell lung cancer (NSCLC) patients harboring oncogenic mutations in the epidermal growth factor receptor (EGFR) inevitably develop resistance to targeted EGFR tyrosine kinase inhibitors (TKI) therapy. To support malignant features associated with cancer development and therapy resistance, the cancer cells adapt their metabolic rate and pathways. As an example, aerobic glycolysis, where the cells use glycolysis in the presence of oxygen, is frequently seen. Here we show that targeting aerobic glycolysis represents a promising strategy in cancer therapeutics.

**Abstract:**

Increased glycolytic activity is a hallmark of cancer initiation and progression and is often observed in non-small cell lung cancer (NSCLC). Pyruvate dehydrogenase (PDH) complex acts as a gatekeeper between glycolysis and oxidative phosphorylation, and activation of PDH is known to inhibit glycolytic activity. As part of a standard therapeutic regimen, patients with NSCLC harboring oncogenic mutations in the epidermal growth factor receptor (EGFR) are treated with EGFR tyrosine kinase inhibitors (EGFR TKIs). Independent of good initial response, development of resistance to this therapy is inevitable. In the presented work, we propose that inhibition of glycolysis will add to the therapeutic effects and possibly prevent development of resistance against both EGFR TKIs and ionizing radiation in NSCLC. Analysis of transcriptome data from two independent NSCLC patient cohorts identified increased expression of pyruvate dehydrogenase kinase 1 (PDHK1) as well as upregulated expression of genes involved in glucose metabolism in tumors compared to normal tissue. We established in vitro models of development of resistance to EGFR TKIs to study metabolism and determine if targeting PDHK would prevent development of resistance to EGFR TKIs in NSCLC cells. The PDHK1 inhibitor dichloroacetate (DCA) in combination with EGFR TKIs and/or ionizing radiation was shown to increase the therapeutic effect in our NSCLC cell models. This mechanism was associated with redirected metabolism towards pyruvate oxidation and reduced lactate production, both in EGFR TKI sensitive and resistant NSCLC cells. Using DCA, the intracellular pool of pyruvate available for lactic fermentation becomes limited. Consequently, pyruvate is redirected to the mitochondria, and reinforces mitochondrial activity. Addition of DCA to cell culture deacidifies the extracellular microenvironment as less lactate is produced and excreted. In our study, we find that this redirection of metabolism adds to the therapeutic effect of EGFR TKI and ionizing radiation in NSCLC.

## 1. Introduction

Drug resistance remains a major challenge in the clinical practice in non-small cell lung cancer (NSCLC) treatment. Development of resistance illustrates an example of cancer cell plasticity and can be regarded as evolution by natural selection. Cancer cells, in a comparable way as organisms, can adapt to environmental changes within the body. In a cellular context this may include hypoxia, nutrient availability, pH and cancer therapy [1]. As a consequence of the latter, cells harboring intrinsic mutations or that acquires favorable mutations are likely to adapt to treatment [2]. One of the essential parts of the “survival of the fittest cancer cell” is metabolic plasticity and rewiring, which provides precursors for biosynthesis and facilitate proliferation. In this study, we investigate metabolic rewiring in acquired drug resistance in epidermal growth factor receptor (EGFR) mutated NSCLC. We show that by inhibition of the pyruvate dehydrogenase kinase 1 (PDHK1), a regulator of the key metabolic checkpoint pyruvate dehydrogenase complex (PDH), the therapeutic effect is significantly increased. 

According to the GLOBOCAN 2020 age-standardized estimates, when both genders are combined, lung cancer is the second most commonly diagnosed cancer, attributing to approximately 18% of the estimated 9.9 million cancer-related deaths [3,4]. Resistance to treatment represents a major cause of the high mortality in lung cancer. Lung cancer is divided into two histotypes: small cell lung cancer (SCLC) and non-small cell lung cancer (NSCLC). NSCLC accounts for more than 80% of the cases and is further divided into subtypes including adenocarcinoma (LUAD) and squamous cell carcinoma (LUSC) [5]. Multimodal treatment regimens are tailored to the patients and vary depending on factors including cancer subtype, performance status, age, grade, tumor location and molecular characteristics [6]. Treatment options include chemotherapy, radiotherapy, surgery, immune and targeted therapy [7,8,9]. Targeted therapies such as tyrosine kinase inhibitors (TKIs) are used in several oncogene-addicted cancers, including NSCLC. EGFR represent a therapeutic target as it has been found to drive oncogenesis in multiple solid cancers [7,9,10]. In NSCLC harboring activating mutations in EGFR, EGFR TKIs have proven to prolong patient survival [6,11]. However, although initially effective, acquired resistance to EGFR TKIs is unavoidable [6]. How to avoid resistance and prolong therapeutic efficacy is a central question to provide new clinically relevant strategies.

Common resistance mechanisms in NSCLC include additional mutations in the EGFR gene [12] or amplification of alternative oncogenes, such as c-MET [13]. Further, phenotypic shifts such as epithelial to mesenchymal transition (EMT) is also recognized as a resistance mechanism. EMT induces both morphological and genetic alterations that may induce resistance to EGFR TKIs [6,12,13]. It has been shown that resistance to the third generation EGFR inhibitor osimertinib is associated with an EMT phenotype, further indicating that EMT is a general resistance mechanism against different generations of EGFR TKIs [14]. Intracellular metabolic alterations have been observed upon treatment with EGFR TKIs [15,16,17] and provides a rationale for investigating if targeting tumor metabolism adjuvant or concurrently with EGFR TKIs would improve therapeutic efficacy.

The capability of malignant cells to deregulate cellular energetics was included in Hanahan and Weinberg’s *Hallmarks of Cancer* [18], highlighting the potential of targeting cancer cell metabolism. Altered metabolic signatures during cancer development was described in 1924 by Otto Warburg in a seminal publication where it was shown that cancer cells preferentially use glycolysis for ATP production even under aerobic conditions. This phenomenon, often referred to as the Warburg effect or aerobic glycolysis takes part in metabolic reprogramming in many cancers [19,20,21,22,23,24]. Metabolic shifts like aerobic glycolysis do not only affect ATP generation, but also increases the biosynthesis of carbohydrates, lipids, proteins and nucleic acids, all of which are crucial building blocks for proliferative cancer cells [22,23,25]. Unphosphorylated PDH (e.g. induced by inhibition of PDHKs) is actively converting pyruvate to acetyl-CoA in the mitochondria, fueling oxidative phosphorylation (OXPHOS). Inhibitory phosphorylation of PDH by the PDHKs leads to increased flux of pyruvate into lactate, by lactate dehydrogenase (LDH) [26,27]. Reduced conversion of pyruvate to lactate deacidifies the microenvironment and reduces the glycolytic activity by a negative feedback loop [28]. Dichloroacetate (DCA), a natural occurring pyruvate analog, acts as a PDHK inhibitor, resulting in increased mitochondrial pyruvate oxidation [29,30]. As a consequence, the production of lactate from pyruvate is decreased and acidification of the microenvironment is reduced [31].

The PDHK1 and PDHK3 have been shown to be regulated by hypoxia inducible factor alpha (HIF1α) as their promoter contain a HIF responsible element (HRE), which allows HIF1α to bind with high affinity [32]. PDHK2, however, has lower affinity for HIF1α and PDHK4 has no affinity for HIF1α. PDHK4 has been shown to be upregulated in response to high fat diets and diabetes [32,33,34,35,36,37]. It has previously been shown that deregulation of PDH and the pyruvate oxidation pathway are involved in tumor initiation and development of cancer [27,38,39,40]. In the presented study, we analyzed expression data from NSCLC patient cohorts to elucidate the possible correlation between PDHK1 and genes involved in key metabolic pathways.

We have investigated the potential of increasing the therapeutic effect in NSCLC cells by manipulation of the pyruvate metabolism. By addition of DCA, we show that the cells increase oxidation of pyruvate and that lactate secretion is massively reduced. Both EGFR TKI sensitive as well as EGFR TKI resistant cells show additional therapeutic effects upon DCA addition. Based on our results, we propose that manipulation of the pyruvate metabolism may add to the therapeutic efficacy of EGFR TKIs and/or ionizing radiation for NSCLC patients. 

## 2. Materials and Methods

### 2.1. Expression Analysis and Correlation Analysis

We investigated expression levels and association between PDHK1 and genes involved in glucose metabolism, gluconeogenesis, fatty acid oxidation and ROS defense. This was done by analyzing RNA sequencing cohorts from lung adenocarcinoma obtained from the cancer genome association (LUAD-TCGA, *n* = 1016), normal lung tissue obtained from TCGA (*n* = 110) and microarray data obtained from the GSE18842 cohort consisting of LUAD and LUSC samples [41]. In the GSE18842 cohort, the normal samples are paired to the cancer samples. The expression level data are presented as relative arbitrary units or FKPM log2 Fold change values. In the correlation values were displayed as Spearman Rho values. All *p*-values from the represented values are provided in Supplementary Table S1.

### 2.2. Cell Culture

All cell lines were cultured at 5% CO_2_ and 21% O_2_ at 37 °C. The non-small cell lung cancer (NSCLC) cell line HCC827 (CRL-2868, ATCC Manassas, VA, USA) were cultured in RPMI-1640 (R8758, Sigma-Aldrich, St. Louis, MO, USA) supplemented with 10% fetal bovine serum (FBS, F-7525, Sigma-Aldrich), 1% L-glutamine (G7513, Sigma) and 50 µg/mL penicillin/streptomycin (P-0781, Sigma). The H1975 (NCI-H1975, CRL-5908, ATCC) cell line was cultured with 5% FBS, otherwise maintained in the same way as HCC827. The erlotinib resistant clone (HCC827/BERL) has developed acquired resistance through in vitro exposure to 1 µM erlotinib over a period of time. H1975 cells and the rociletinib (CO-1686) resistant clone (COR1-1) were provided by Clovis Oncology (Boulder, CO, USA) [42]. The resistant clones were continuously cultured with 1 µM of the respective EGFR TKI in the medium. Both cell lines were authenticated by using the ATCC human STR Cell Authentication Service and regularly tested for mycoplasma by using the Mycoalert kit from Lonza (LT07-705, Basel, Switzerland). 

All compounds were titrated before use. 20 mM dichloroacetate (DCA, 347795, Sigma-Aldrich), 1 µM erlotinib (5083 Cell Signaling, Leiden, The Netherlands), 1 µM rociletinib (CO-1686, Clovis oncology) was used if otherwise not stated. All data normalization was done by protein measurements through the Pierce BCA Protein Assay Kit (Thermo Fisher Scientific, Waltham, MA, USA) if otherwise not stated.

### 2.3. qPCR

RNA was extracted through the use of RNeasy Mini Kit (74104, QIAGEN, Venlo, The Netherlands), including DNA digestion, and cDNA was made by the High-Capacity cDNA Reverse Transcription Kit (4368813, Thermo Fisher Scientific)) according to manufacturer instructions. The qPCR was run with 3 replicates of 5 µL in the Light Cycler 480 system (Roche, Basel, Germany), and 3 replicates of 1 µL as described in Dyrstad et al., 2018 [43]. mRNA expression was normalized to eukaryotic 18s expression (4352930E) and calculated based on the fold change (2^-∂∂Ct^) method [44]. The probes used were all from Applied Biosystems (Thermo Fisher Scientific). List of probes were as follows: E-CADHERIN (CDH1, HS01023894), N-CADHERIN (CDH2, HS00983056), LDHA (HS01378790), LDHB (HS00929956), PGC1α (PPARGC1A, HS01016719) and VIM (HS00185584). 

### 2.4. Drug Response Curves and Cell Proliferation

Cells were plated at a density of 3000 cells/well in 96-well Nunclon Delta Surface plates (Thermo Fisher Scientific). Following overnight incubation, cells were treated with either DCA, erlotinib, rociletinib or DCA in combination with erlotinib or rociletinib. After radiation and/or after treatment the 96well plates were placed in a Incucyte^®^ Live Cell Analysis system (Incucyte ZOOM 2016B, EssenBioscience Ltd., UK) and percent confluency was estimated using the Incucyte Zoom software and normalized to the first time point of each cell type or DMSO control as described in figure legends. The resazurin cell viability assay was used to measure cell viability by using fluorescence to detect cellular metabolic activity. After indicated treatment period (72 h), resazurin was diluted in medium (10% *v*/*v*) and added to the cells. The cells were further incubated for 3 h before fluorescence was measured using a Tecan SPARK instrument (Tecan Trading AG, Switzerland).

### 2.5. Clonogenic Assay

2500 (H1975) or 7500 (HCC827) cells were plated in 6w plates (Nunc, Thermo Fisher Scientific) and treated alone or in combination with 20 mM DCA and 1 µM erlotinib or 1 µM rociletinib as indicated on the figure. After 14 days, they were fixed with a mixture of 6% (*v*/*v*) glutaraldehyde and 0.5% (*v*/*v*) crystal violet. Cells were imaged with an EPSON Perfection V850 Pro scanner (Epson, Suwa, Japan).

### 2.6. Western Blotting 

Cells for protein extraction were grown in T75 flasks, washed with cold PBS, scraped, pelleted and frozen in −80 °C, before they were lysed using RIPA lysis buffer. 20 µg protein was loaded into 6–12% SDS-PAGE gels from Bio-Rad Laboratories (Hercules, CA, USA). Blotting was performed by using the Bio Rad Trans-Blot Turbo system and blocking was performed for 2 h in 5% milk in TBS-T (0.1% (*v*/*v*) Tween). Primary antibodies used were PDK1 (ab207450, Abcam, Cambridge, UK), and p-PDH (ab92696, Abcam), E-Cadherin (CDH1, 14472S, cell signaling), vimentin (VIM, ab92547, Abcam), LDHA (3582, Cell signaling). The housekeeping protein a-tubulin or vinculin was used as loading control when total protein staining was not available. Primary antibodies were removed, and the membranes were washed three times for 5 min with TBS-T before incubating them with secondary antibody for 1 h under agitation. The membranes were washed three times with TBS-T before they were examined. Complete blots can be found in Supplementary Figure S4.

### 2.7. Immunocytochemistry

For immunostaining, HCC827 and H1975 cells were plated on coverslips in 24 well plates (Nunc) and cultured until they reached 70% confluency. Fixation and permeabilization was done by adding 37% PFA and TBS-T, and staining was done by with primary antibodies for E-cadherin (cat # 14472, Cell Signaling, Leiden, The Netherlands) and vimentin (AB92547, Abcam, UK), which were diluted 1:100 in TBS-T with 0.5% BSA. The secondary antibodies used were Alexa 594 anti-mouse and Alexa-647 anti-rabbit. Phalloidin AF555 (a34055, Thermo Fisher Scientific) was diluted 1:40 and used according to the manufacturer’s instructions. The coverslips were mounted with Prolong Dimond with Dapi (Thermo Fisher Scientific). Images were acquired by using a Leica TC2 SP8 STED 3X microscope with HC PL APO CS2 lasers using the 100 × 1.4 NA oil objective as described in Røsland et al., 2019 [45].

### 2.8. Oxygen Consumption

The Seahorse XFe96 Analyzer (Agilent, Santa Clara, CA, USA) was used to measure oxygen consumption rate (OCR) and extracellular acidification rate (ECAR). The mitostress and glycostress tests were performed as described in [35,45,46]. Cell number and concentration of compounds was optimized before running the assay. All compounds were from Sigma-Aldrich if otherwise not stated. The mitostress assay medium used was D5030 supplemented with 10 mM glucose, 2 mM pyruvate and 4 mM glutamine at pH 7.4 (+/− 0.05). Throughout the mitostress experiments, we used 3 µM oligomycin, 0.5 µM CCCP (HCC827) or 1.5 µM CCCP (H1975), 1 µM rotenone and 1 µM antimycin A. Data was rox (antimycin A, non-mitochondrial respiration) corrected. When measuring glycolysis, the assay medium contained 4 mM glutamine and pH 7.35 (+/− 0.05). 10 mM glucose was added in port A, oligomycin port B, CCCP port C and 100 mM 2DG in port D. Upon investigating the acute effects of DCA, 20 000 cells were plated the day before the assay in Seahorse 96 well plates. Mitostress assay medium was used. The pH of the DCA addition was carefully adjusted prior to loading. DMSO control, 20 mM DCA, 1 µM EGFR TKI or a combination of DCA and EGFR TKI was added to port A, oligomycin in B, CCCP in port C and rotenone and antimycin A in D. For 16 h DCA treatment, at day 1, 5000 cells were plated in Seahorse 96 well plates and left to set overnight. At day 2, 20 mM DCA was added to the culture medium. The third day, the run was performed as a mitostress test. Data was normalized by using the Pierce BCA Protein Assay Kit (Thermo Fisher) and protein concentration was calculated by using the associated BSA standard curve.

### 2.9. CO_2_-Trapping

Substrate oxidation rate of the NSCLC cells was based on [47] and performed as described in [35] without modification. The assay medium contained Dulbecco’s phosphate-buffered saline (with MgCl_2_ and CaCl_2_, #D8662) with 10 µM added fatty acid free BSA as assay medium. The substrates used were [U-^14^C] labeled glucose (2 µCi/mL), [1-^14^C]-pyruvate (0.25 µCi/mL) and [U-^14^C]-lactate (0.5 µCi/mL) all from PerkinElmer (Waltham, MA, USA). The glucose assay medium contained a total of 5 mM glucose, the pyruvate assay medium contained a total of 200 µM pyruvate and 0.5 mM glucose and the lactate medium contained a total of 1 mM Lactate.

For untreated cells, 30,000 cells were plated overnight in a 96-well Corning CellBIND Surface Polystyrene plate (Corning, NY, USA). For the DCA treated cells, 10,000 (HCC827) and 7500 (H1975) cells were plated and incubated overnight, before 20 mM DCA or control medium was added to the cells and left for 24 h before trapping. On the day of trapping, the ^14^C-labeled medium was added to the cells before a filter plate immersed with 1 M NaOH and a silicon gasket were placed on top and clamped in a sandwich. The “sandwich” was then incubated for 4 h. After incubation, cells were washed twice with PBS before they were lysed with 0.1 M NaOH. Protein was measured using the Pierce BCA Protein Assay Kit as described by manufacturer. 30 µL MicroScint scintillation liquid (Perkin Elmer) was added to each well in the filter plate and incubated for one day before reading the accumulated ^14^CO_2_ trapped in the filters using top read on the MicroBeta Microplate Counter (Perkin Elmer). All numbers were subtracted from background medium control levels. If substrate levels were negative (below background) they were set to 0.

### 2.10. Ionizing Radiation

The radiation was performed at Haukeland University Hospital by a Varian Triology (Energy 6 MV, 600 MU/min ~1.3 Gy/min). Cells were plated in 96w plates (cell proliferation) and incubated over night; cell number was optimized to treatment length. The cells were exposed to 0 Gray or 8 Gray, with gel-covers mimicking the density in the normal human thorax. After radiation the cells were treated alone or in combination of 20 mM DCA and 1 µM erlotinib.

### 2.11. Statistics and Figures

Data were analyzed using Graphpad Prism 8 software (Graph-Pad Software; San Diego, CA, USA) and figures made by using Adobe Illustrator (San Jose, CA, USA). All experiments were repeated at least three times and representative experiments are shown either as mean +/− SD or mean +/− SEM. ANOVA and unpaired two-tailed student’s t-test were used to evaluate statistical differences between the samples. Rho values >0.3 and <−0.3 were considered significant. *p* < 0.05 was considered statistically significant.

## 3. Results

### 3.1. Increased PDHK1 Expression Is Associated with Altered Expression of Genes Involved in Glucose Metabolism in NSCLC Human Tumors

**Figure 1:**
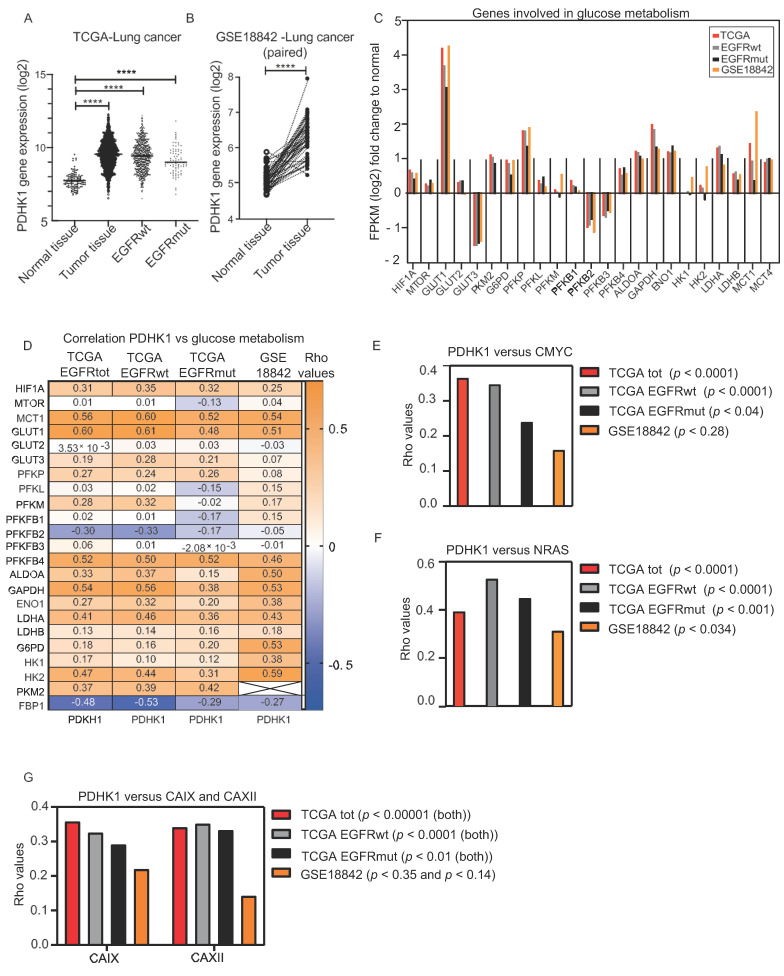
Gene expression and correlation analysis of PDHK1 and genes involved in glucose metabolism in non-small cell lung cancer (NSCLC) tumors. The expression level of selected genes was investigated in two individual transcriptomic NSCLC cohorts. (**A**) PDHK1 RNA sequencing data from the cancer genome atlas (TCGA) (*n* = 110 adjacent normal tissue sample, *n* = 1016 LUAD or LUSC samples) in the total cohort, and in EGFR wild-type (EGFRwt) and EGFRmutated (EGFRmut) samples, and (**B**) PDHK1 mRNA microarray data from paired samples from GSE18842 (*n* = 44 adjacent normal tissue samples and *n* = 46 LUAD or LUSC samples). (**C**) Relative expression (FPKM log2 fold change) for a panel of genes involved in glucose metabolism. (**D**) Correlation analysis (Spearman Rho values) between PDHK1 and the panel of genes involved in glucose metabolism. Correlation analysis (Spearman Rho values) of (**E**) PDHK to c-MYC, (**F**) NRAS and (**G**) CAIX and CAXII. *p* values are presented for all the included data in Supplementary Table S1. Unpaired two-tailed t-test was used for (A) **** *p* < 0.0001.

We investigated PDHK1 expression levels and correlated the PDHK1 levels to a panel of genes involved in glucose metabolism in two independent cohort datasets from TCGA and GSE18842 with LUAD, LUSC and normal tissue samples. PDHK1 mRNA level was significantly higher in cancer tissue compared to normal counterpart in both cohorts. In the TCGA EGFR wild type group (EGFRwt, *n* = 591), the upregulation was more prominent, compared to the group with EGFR mutations (EGFRmut, *n* = 76) (Figure 1A). Additionally, in the GSE18842 cohort representing paired samples (both tumor samples and normal tissue from the same patient), the upregulation of PDHK1 was prominent in tumor compared to normal tissue (Figure 1B). To evaluate effects on glucose catabolism in tumors relative to normal tissue, we investigated expression levels of 22 related genes, both regulators and enzymes. The majority of these genes showed significant upregulation in cancer tissue compared to normal tissue (Figure 1C). In particular, the expression of glucose transporter GLUT1 was increased, consistent with increased glucose uptake frequently found in growing tumors. 

Furthermore, multiple glycolytic enzymes were found to be increased, including PKM2 (only available data from the TCGA cohort), G6PD, PFKP, ALDOA, GAPDH, ENO1, LDHA, and the lactate importer MCT1. For most of the genes, these findings were consistent in both cohorts, and were generally similar in EGFRwt compared EGFRmut tumors. Correlation analysis between PDHK1 and the panel of genes involved in glucose metabolism revealed significant positive associations for a majority of genes (Rho values >+0.3, *p* < 0.05) (Figure 1D). This indicates that PDHK1 is co-regulated with the glycolytic machinery at the transcriptional level. Further, in this case, the EGFRwt and EGFRmut groups showed similar results for the majority of the genes. Moreover, two key HIF1α regulated genes involved in tumor acid/base regulation are the hypoxia-regulated carbonic anhydrases CAIX and CAXII, which catalyze the reversible hydration of CO_2_ to HCO_3_^−^ + H^+^. Increased expression of these proteins can lead to exacerbated intracellular alkalization and extracellular acidification. Genes encoding for both CAIX and CAXII were found to correlate to the expression of PDHK1 in our dataset (Figure 1G).

The EGFRmut group tended to display lower Rho values, which may be biologically relevant, though it could also result from the smaller sample size for this group. Interestingly, the oncogene c-MYC, known to interact with HIF1α to enhance glycolysis in cancer cells [48], was correlated to the expression of PDHK1 in our data. However, only a trending association was observed in EGFRmut and the GSE18842 cohort (Figure 1D). Additionally, the oncogene NRAS has been shown to be a regulator of HIF1α and glycolysis [49], and it was also correlated to the expression of PDHK1 in our data (Figure 1E). We found genes involved in gluconeogenesis to be downregulated in tumor samples compared to normal tissue samples in both cohorts (Supplementary Figure S1A). The level of fructose bisphosphatase 1 (FBP1), a key regulator of gluconeogenesis activity was particularly decreased in tumors relative to normal tissue, and interestingly, as seen in Supplementary Figure S1A, mRNA levels of FBP1 and PDHK1 were inversely correlated. This correlation was more prominent in the EGFRwt group compared to the EGFRmut group, which may indicate that the metabolic changes during tumor development are different in the two groups. Furthermore, genes involved in FAO including ACOX4, CYP4A11, PPARG and PDHK4 were generally downregulated in NSCLC compared to normal tissue (Supplementary Figure S1B). Interestingly, PDHK1 was inversely correlated to PDHK4 (Supplementary Figure S1C), suggesting distinct roles of the two enzyme family members.

Expression of several genes encoding antioxidant enzymes were upregulated in NSCLC compared to normal tissue (Supplementary Figure S1D). Three genes of the glutathione peroxidase (GPX) family were upregulated, particularly GPX2, and to a lesser extent GPX7 and 8. GPX3 was significantly downregulated. The thioredoxins TXN and TXNRD1 were upregulated in the total TCGA cohort and in the EGFRwt group, but less in the EGFRmut group, suggesting differential regulation in cancer cells harboring EGFRwt compared to EGFRmut group. For the peroxiredoxin family, 4 out of 6 genes were upregulated in the two cohorts. Moreover, the tumor mRNA expression of glutathione S-reductase (GSR) and superoxide dismutase 1 (SOD1) showed a trend of increase, whereas catalase (CAT) was significantly downregulated. The TCGA data showed slightly reduced expression of the mitochondrial SOD2 in the tumors. Interestingly, the mRNA level of multiple antioxidant genes correlated with PDHK1 level (Supplementary Figure S1E). For this set of genes, PDHK1 predominantly displayed positive associations, such as for GPX2, GPX7 and TCNRD1. However, negative associations were observed, such as for GPX3 and TXBRD2. These data support the notion that regulation of PDHK1 may be associated with mechanisms of ROS defense.

### 3.2. DCA Add to the Inhibitory Effect of Cell Growth by EGFR TKIs in NSCLC Cell Models

**Figure 2:**
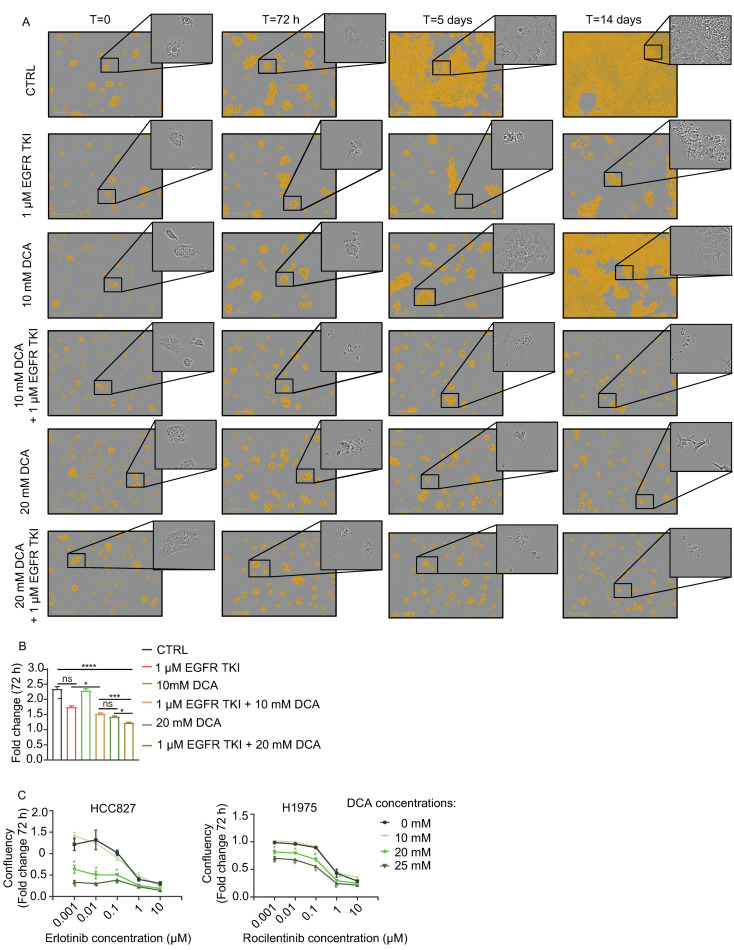
Antiproliferative effects of dichloroacetate (DCA) in combination with epidermal growth factor receptor (EGFR) tyrosine kinase inhibitors (TKIs) in NSCLC cell models. (**A**) Microscopy of HCC827/PAR cells treated with 1 µM erlotinib, 10 or 20 mM DCA as single treatment or in combination with 10 mM/20 mM DCA. The images show representative cultures at T = 0 h, T = 72 h, T = 5 days and T = 14 days. (**B**) Quantified proliferation data for HCC827/PAR cells at 72 h from the experiment described in (**A**). The significance between the treatments were **** if otherwise not indicated on the figure. (**C**) Dose dependent effects of EGFR TKI combined with DCA, in HCC827/PAR and H1975/PAR cell cultures. In the right panel, * indicate significant (*p* < 0.05) difference from erlotinib treated 0 mM DCA treated control and data is displayed as mean +/− SEM. Representative experiment (1/3) with 3–6 technical replicates. (Statistical analysis in (**B**) was performed by a one-way ANOVA, with Tukey post hoc test. (**C**) was performed by two-way ANOVA with Tukey post hoc test. * *p* < 0.05, ** *p* < 0.01, *** *p* < 0.001, **** *p* < 0.0001.

Based on the finding that NSCLC tumors had upregulated PDHK1, we investigated effects of the PDHK inhibitor DCA on cells, either alone or in combination with EGFR TKI. We used HCC827, a NSCLC cell line sensitive to the first-generation EGFR TKI erlotinib. In addition, we used H1975 cells which are resistant to erlotinib, but sensitive to the third generation EGFR TKI rociletinib. Microscopy analysis showed a time-dependent inhibition of proliferation and increased cell death in HCC827 cultures treated with both erlotinib and DCA during a period of 14 days, with only cell debris remaining at the end of the treatment period (Figure 2A, lower right corner). After 72 h, a potentiated effect of the erlotinib + 20 mM DCA treatment was shown, compared to either of the two agents alone (Figure 2B). When treated with one of the agents, reduced cell number was observed, but the majority of the remaining cells appeared intact and viable. In parallel, different dosage combinations of the agents were performed to measure confluence and resazurin viability (Figure 2C and Supplementary Figure S2). Both cell models displayed a similar pattern after 72 h treatment. An increased antiproliferative effect of 20 or 25 mM DCA in combination with erlotinib/rociletinib was observed. A concentration of 1 µM was required to give a significant effect of the EGFR TKIs monotherapy, but additional effect of DCA was also found at lower concentration of the EGFR TKIs. These data demonstrate that DCA in NSCLC cell inhibits cell proliferation as a single agent and adds to the therapeutic effect of EGFR TKI for both HCC827 and H1975 cells.

### 3.3. Acute Metabolic Effects of DCA in NSCLC Cell Models

**Figure 3:**
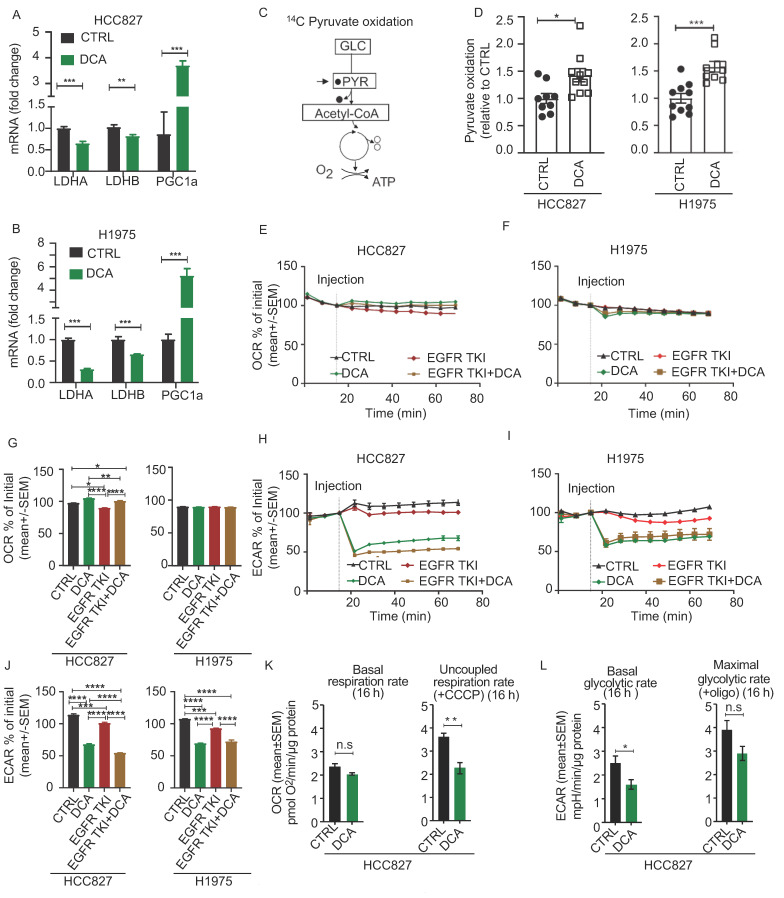
Acute effects of DCA on NSCLC cell metabolism. The acute effect of DCA on cell metabolism was evaluated by measuring related mRNA levels of LDHA, LDHB and PGC1α in HCC827 (**A**) and H1975 (**B**) cells. The column diagrams represent relative mRNA expression of LDHA, LDHB and PGC1α from one of three experiments in HCC827 and H1975 cells treated with 20 mM DCA for 48 h and is represented as mean +/− SD. (**C**) Graphic illustration showing metabolic entry and release of 14C labelled carbons (filled circles) in the 14C-pyruavte oxidation assay. The [1–14C] pyruvic acid were used, and complete oxidation was measured by quantifying the produced ^14^CO_2_. Following treatment with 20 mM DCA for 24 h we measured cellular pyruvate oxidation (**D**). The data in these experiments represent fold change pooled from two experiments (*n* = 8–10 per experiment, data represented as mean +/− SEM). (**E**,**F**) shows representative real-time OCR traces obtained from HCC827/PAR cells (mean +/− SEM) (**E**) and H1975/PAR (**F**). OCR is calculated as percent of initial rate measured immediately prior to addition of DCA. (**G**) Column diagrams comparing DCA and/or EGFR TKI induced effects on OCR in HCC827 and H1975, 54 min after adding drug. The statistics are based on this representative run (1/3 in total), where the technical replicates range from 5–11 per experiment (+/− SEM). Representative real-time ECAR traces obtained from HCC827/PAR cells (**H**) and H1975/PAR (**I**) Data are represented as mean +/− SEM. (**J**) Column diagrams comparing DCA and/or EGFR TKI induced effects on ECAR in HCC827 and H1975, 54 min after adding drug. The statistics are based on this representative run (1/3 in total), where the technical replicates range from 5–11 per experiment. (**K**) Basal oxygen consumption rate (OCR) and uncoupled OCR (after addition of CCCP) are presented from a representative experiment where cells were treated 16 h with 20 mM DCA in culture medium in HCC827/PAR cells. The statistics are based on this representative run (1/2 in total), where the technical replicates range from 5–11 per experiment. Data are represented as mean +/− SEM. (**L**) Extracellular acidification rate (ECAR) reflecting basal glycolysis and maximal glycolysis (after addition of oligomycin) from the same experiment as in (**K**). Statistical analysis was performed by two-way ANOVA with Tukey post hoc test for (**G**) and (**J**), and an unpaired two-tailed t-test for (A,B,D,K–L) (* *p* < 0.05, ** *p* < 0.01, *** *p* < 0.001, **** *p* < 0.0001).

DCA is known to promote mitochondrial pyruvate oxidation by activation of the PDH enzyme complex, resulting in reduced LDH activity. We found decreased LDHA and LDHB mRNA levels in both cell lines after treatment with 20 mM DCA for 48 h. In addition, the mRNA level of PGC1α was strongly upregulated in DCA treated cells in both cell lines, supporting metabolic adaptations involving increased mitochondrial biogenesis (Figure 3A,B). Further, to confirm that DCA treatment redirects pyruvate flux towards mitochondrial oxidation in our model, we incubated cells with ^14^C-labeled version of pyruvate and measured production of ^14^CO_2_ resulting from the oxidative decarboxylation catalyzed by PDH (Figure 3C). We found increased rates of pyruvate oxidation after treatment with DCA in both cell models (Figure 3D). Further, we measured oxygen consumption rate (OCR) and extracellular acidification rate (ECAR) to study the acute effect of mitochondrial and glycolytic lactate production upon addition of DCA. Addition of DCA, alone or in combination with EGFR TKI, resulted in a small, but significant increase of OCR in HCC827 cells (Figure 3E), but not in the H1975 cell line (Figure 3F). The diagrams in Figure 3G show the statistics from this representative experiment. More profound, ECAR acutely decreased upon DCA addition in both cell lines (Figure 3H,I). The diagrams in Figure 3J are showing the statistics from this experiment. Of note, these effects of DCA were also seen in the presence of EGFR TKI (Figure 3H–J). We ensured that the DCA solution (pH 7.4) did not change the pH of the medium itself by adding DCA solution in wells without cells. In an experiment where we treated the HCC827/PAR with 20 mM for 16 h, we found no change in the basal respiratory capacity, whereas the respiratory capacity was decreased (Figure 3K). The extracellular acidification rate, reflecting the glycolytic activity remained reduced (Figure 3L), confirming that the effect of DCA induced decreased cellular lactate secretion in both an acute and in a long-term manner in our cell models. Together, the functional and regulatory data support that DCA promotes mitochondrial pyruvate oxidation at the expense of lactate production in NSCLC cells. However, increased pyruvate oxidation by DCA does not seem to affect the overall OCR. 

### 3.4. Development of EMT Mediated Resistance to EGFR TKI in NSCLC Cells

**Figure 4:**
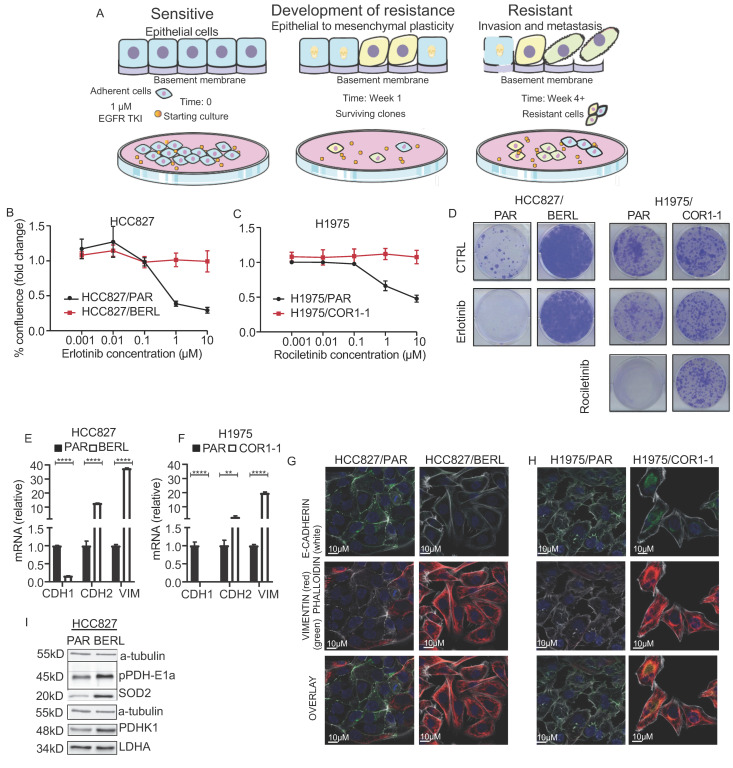
Resistance to EGFR TKI is associated with EMT and increased expression of PDHK1. (**A**) Schematic overview of the in vitro model of development to resistance to EGFR TKI. (**A**) was made using smart servier medical art (http://smart.servier.com (assessed on 20 January 2021)). The effect of EGFR TKIs on cell proliferation was evaluated in resistant cells compared to the respective parental cells. The erlotinib/rocilentinib dose curves display data (mean +/− SEM) for the NSCLC EGFR TKI resistance models: (**B**) HCC827/PAR (parental) and HCC827/BERL (resistant), (**C**) H1975/PAR (parental) and H1975/COR 1-1 (resistant), (A) representative experiment (1/4 in total) with 3–6 technical replicates is shown. (**D**) Clonogenic potential of HCC827/PAR and HCC827/BERL, and H1975/PAR and H1975/COR1-1 upon erlotinib or rociletinib treatment. Relative mRNA expression levels of CDH1 (E-cadherin), CDH2 (N-cadherin) and vimentin in EGFR TKI resistant cells compared to parental cells (HCC827 (**E**) and H1975 (**F**)), data represented as mean +/− SD. Immunostaining and fluorescence microscopy showing expression of E-cadherin, vimentin, and F-actin (Phalloidin) in the two NSCLC EGFR TKI resistance models (HCC827 (**G**) and H1975 (**H**)). (**I**) Western blot analysis of pPDH E1 alpha, SOD2, PDHK1 and LDHA, with α-tubulin as loading control in HCC827 cells. * indicate significant (*p* < 0.05) values. Statistical analysis was performed by unpaired two-tailed t-test (** *p* < 0.01, **** *p* < 0.0001).

We treated HCC827 cells with 1 µM erlotinib for more than 4 weeks to establish an EGFR TKI resistant sub cell line (Illustrating model in Figure 4A). The sub cell line is referred to as HCC827/BERL. In addition, we used the previously established H1975 cell line, with a sub cell line (H1975/COR1-1) resistant to the third generation EGFR TKI rociletinib (CO-1686) [42,50]. The H1975 cell line is known to be resistant towards erlotinib as they have a T790M mutation in EGFR exon 20, but sensitive to rociletinib. By measuring confluence after 72 h of exposure to increasing concentrations of EGFR TKI, we confirmed EGFR TKI resistance in the two cell models (Figure 4B,C). We further studied the clonogenic potential upon treating cells with rociletinib and erlotinib. Clonogenic capacity was absent in the HCC827 EGFR TKI sensitive cell lines treated with erlotinib, whereas H1975 cells were resistant to erlotinib and sensitive to rociletinib. HCC827/BERL were resistant to erlotinib, whereas H1975/COR1-1 were resistant to rociletinib (Figure 4D). By measuring mRNA (Figure 4E,F) and using microscopy and immunocytochemistry (Figure 4G,H), typical features of EMT were documented in EGFR TKI resistant cells, including mesenchymal morphology, low E-cadherin levels and high vimentin levels. The effects on E-cadherin and vimentin protein levels were supported by western blot analysis, and c-MET mRNA was reduced (Supplementary Figure S3B,C). Increased phosphorylation of the PDHK1 E1a subunit and increased PDHK1 protein levels was seen by WB in HCC827/BERL cells (Figure 4I). No changes in protein expression of LDHA were observed upon drug resistance. Finally, expression of SOD2 protein was increased in the erlotinib resistant cells, indicating an alteration of the mitochondrial antioxidant system upon development of resistance to EGFR TKI (Figure 4I). 

### 3.5. Mitochondrial Respiration Is Decreased in NSCLC in EGFR TKI Resistant Cell Models

**Figure 5:**
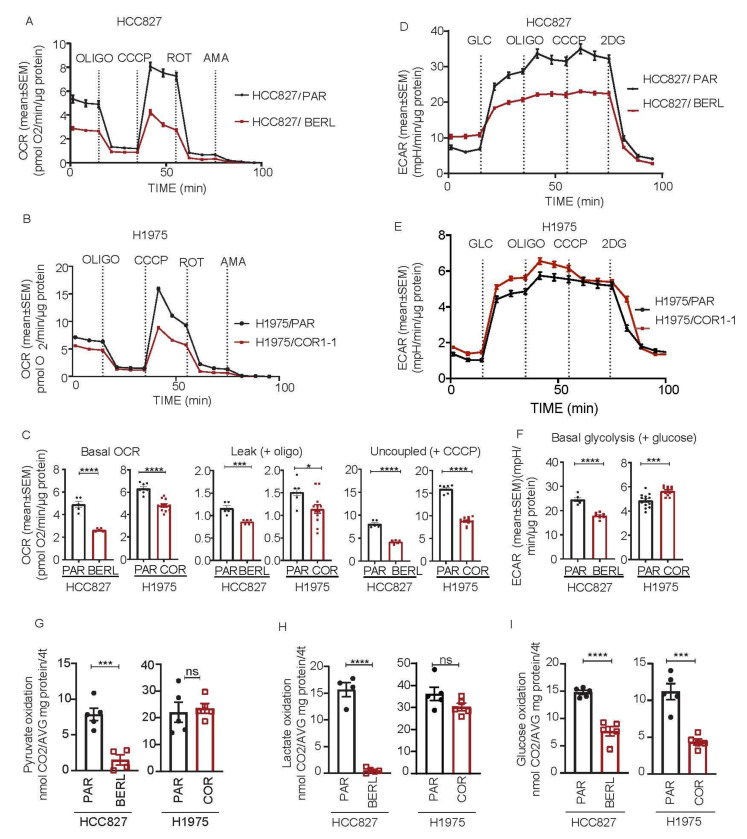
Metabolic phenotyping of parental (PAR) and resistant NSCLC HCC827 and H1975 cells. Oxygen consumption rate (OCR) and extracellular acidification rate (ECAR) was measured in NSCLC cells sensitive and resistant to EGFR TKI. All seahorse OCR data was adjusted for non-mitochondrial activity. (**A**) OCR traces of HCC827/PAR and HCC827/BERL, (**B**) OCR traces of H1975/PAR and H1975/COR1-1, all with sequential addition of oligomycin (3 µM), CCCP (0.5 µM HCC827 and 1.5 µM H1975), rotenone (1 µM) and antimycin (A) (1 µM) as indicated on the figures. (**C**) Column diagrams showing basal respiration, leak proportions and uncoupled respiration rates in HCC827 and H1975. ECAR traces of HCC827/PAR and HCC827/BERL (**D**) and H1975/PAR and H1975/COR1-1 (**E**) both with sequential addition of glucose (10 mM), oligomycin (3 µM), CCCP (0.5 µM HCC827 and 1.5 µM H1975) and 2-DG (100 mM). (**F**) Column diagrams showing basal glycolytic rates in HCC827 and H1975. Substrate oxidation rates in HCC827 and H1975 parental and EGFR TKI resistant cells, (**G**) Pyruvate oxidation rates, (**H**) Lactate oxidation rates and (**I**) Glucose oxidation rates. Data are displayed as a mean +/− SEM of *n* = 5–13 from a representative run (1/3 in total). Statistical analysis was performed by an unpaired two-tailed t-test (* *p* < 0.05, *** *p* < 0.001, **** *p* < 0.0001).

To determine if development of EGFR TKI resistance affects mitochondrial function in NSCLC cells, we measured respiratory rates following sequential addition of specific modulators. For each cell model, the resistant sub cell lines revealed significantly lower rates of mitochondrial respiration when compared to the respective parental cells (Figure 5A,B). This effect was found both for basal respiratory activity and uncoupled maximal respiratory capacity. The leak respiration upon oligomycin addition was normal or low, indicating that the integrity of the inner mitochondrial membrane is intact. Statistics for basal respiration, leak respiration and uncoupled maximal respiration are shown in Figure 5C. HCC827/BERL cells had lower basal ECAR after adding glucose, and maximal ECAR in presence oligomycin, compared to the parental cells, reflecting decreasing glycolytic activity upon development of resistance (Figure 5D). In contrast, H1975/COR1-1 had increasing ECAR levels compared to the parental cells, indicating an increased glycolytic rate upon development of resistance (Figure 5E). Statistics for glycolytic activity are shown in Figure 5F. The resistant HCC827/BERL cells had lower oxidation rates of pyruvate, lactate and glucose, compared to the parental cells (Figure 5G–I). The resistant H1975/COR1-1 cells exhibited similar oxidation rates of pyruvate and lactate compared to parental cells, whereas the rate glucose oxidation was reduced (Figure 5G–I). These effects on substrate oxidation aligns well with the significant reduction in mitochondrial respiration (Figure 5A,B). 

### 3.6. DCA Disrupt the Metabolic Signature and Decrease Viability in NSCLC Cells Resistant to EGFR TKI Therapy

**Figure 6:**
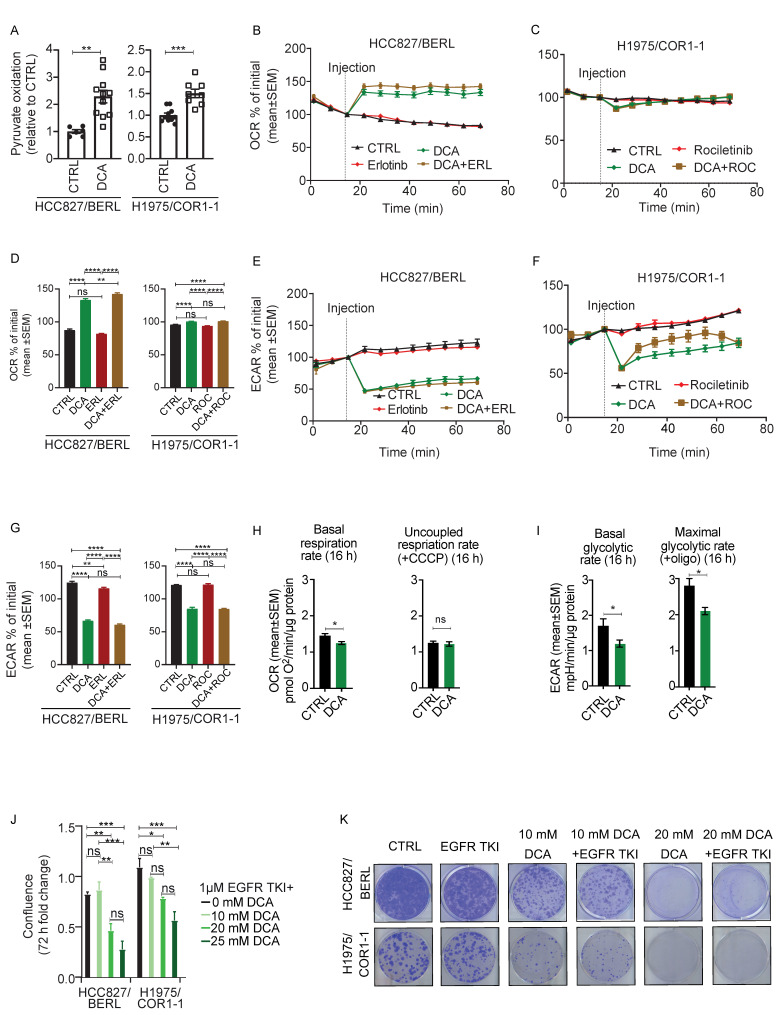
Co-treatment with DCA is adding to the effect of EGFR TKI therapy in NSCLC cells. The acute effect of DCA on cell metabolism was evaluated by measuring pyruvate oxidation, clonogenic potential, oxygen consumption rate (OCR) and extracellular acidification rate (ECAR). The [1–14C] pyruvic acid was used and complete oxidation was measured by quantifying the produced ^14^CO2. (**A**) Following treatment with 20 mM DCA for 24 h, we measured cellular pyruvate oxidation in HCC827/BERL and H1975/COR1-1 clones. Data are displayed as fold change relative to DMSO control as a mean +/− SEM of *n* = 6–10 per experiment with data from 2 representative experiments out of 4 (in total). Real-time OCR and ECAR 54 min after addition of DCA were measured. Representative data of OCR from HCC827/BERL (**B**) and H1975/COR1-1 (**C**). All OCR data were adjusted for non-mitochondrial activity and is displayed as percent +/− SEM of initial (third measurement) prior to addition of control medium, 20 mM DCA or 1 µM erlotinib or 20 mM with 1 µM erlotinib. (**D**) Column diagrams are made from representative data displayed as mean +/− SEM 54 min after addition of DCA. ECAR data under the same conditions are shown for HCC827/BERL (**E**) and H1975/COR1-1 (**F**). (**G**) Column diagrams are made from representative data 54 min after addition of DCA. Basal respiration and uncoupled respiration rates from a representative experiment (1/3) is presented in (**H**) and basal glycolytic rate and maximal glycolytic rate are presented in (**I**) after 16 h treatment with 20 mM DCA in HCC827/BERL. The effect of 1 µM EGFR TKI (erlotinib for HCC827 and rociletinib for H1975) alone or in combination with DCA on cell proliferation was evaluated in resistant cells compared to the respective parental cells in (**J**). In (**K**) the colony formation capacity of the resistant cell lines is shown upon treatment with 10 mM DCA, 10 mM DCA with 1 µM EGFR TKI, 20 mM DCA, and 1 µM EGFR TKI combined with 20 mM DCA as indicated on the figure. Statistical analysis was performed by two-way ANOVA, with Tukey post hoc test in (**A**–**D**), and an unpaired two-tailed t-test for **D**–**I** (* *p* < 0.05, ** *p* < 0.01, *** *p* < 0.001, **** *p* < 0.0001).

To investigate the effect of DCA in EGFR TKI resistant cells, we repeated the previous experiments performed on parental cells (Figure 3). Additionally, for the resistant sub cell lines, increased pyruvate oxidation was observed upon treatment with DCA (Figure 6A). Furthermore, addition of DCA, alone or in combination with EGFR TKI, lead to acute increased OCR in HCC827/BERL cells (Figure 6B), significantly more pronounced compared to the parental cells (Figure 3C). H1975/COR1-1 displayed no change in OCR after DCA addition (Figure 6C). The statistics from the presented experiment are shown in Figure 6D. Further, as seen for the parental cells in Figure 3, exposure to DCA dramatically reduced ECAR in both resistant cell lines (Figure 6E,F). The statistics from the presented experiment are shown in Figure 6G. Upon treating the HCC827/BERL cells with 20 mM DCA for 16 h, the basal respiration rate was decreased, whereas the respiratory capacity (+CCCP) remained unchanged (Figure 6H), confirming that the acute DCA response on OCR were reversed over time. However, the glycolytic rate, as well as the maximal glycolysis (+oligomycin) remained decreased over time (Figure 6I), indicating that DCA triggers a metabolic shift in the EGFR TKI cells.

In combination with 1 µM EGFR TKI, DCA demonstrated a dose-dependent antiproliferative effect on the resistant cells (Figure 6J). Moreover, the colony formation capacity was decreased upon treatment with 10 mM DCA. The clonogenic potential was further decreased upon combining 10 mM DCA with 1 µM EGFR TKI. In both 20 mM DCA, and 1 µM EGFR TKI + 20 mM DCA, the clonogenic potential was totally absent for both EGFR TKI resistant subtypes (Figure 6K).

### 3.7. The Effect of Ionizing Radiation Is Increased in Combination with DCA and EGFR TKI in NSCLC Cells Resistant to EGFR TKI

**Figure 7:**
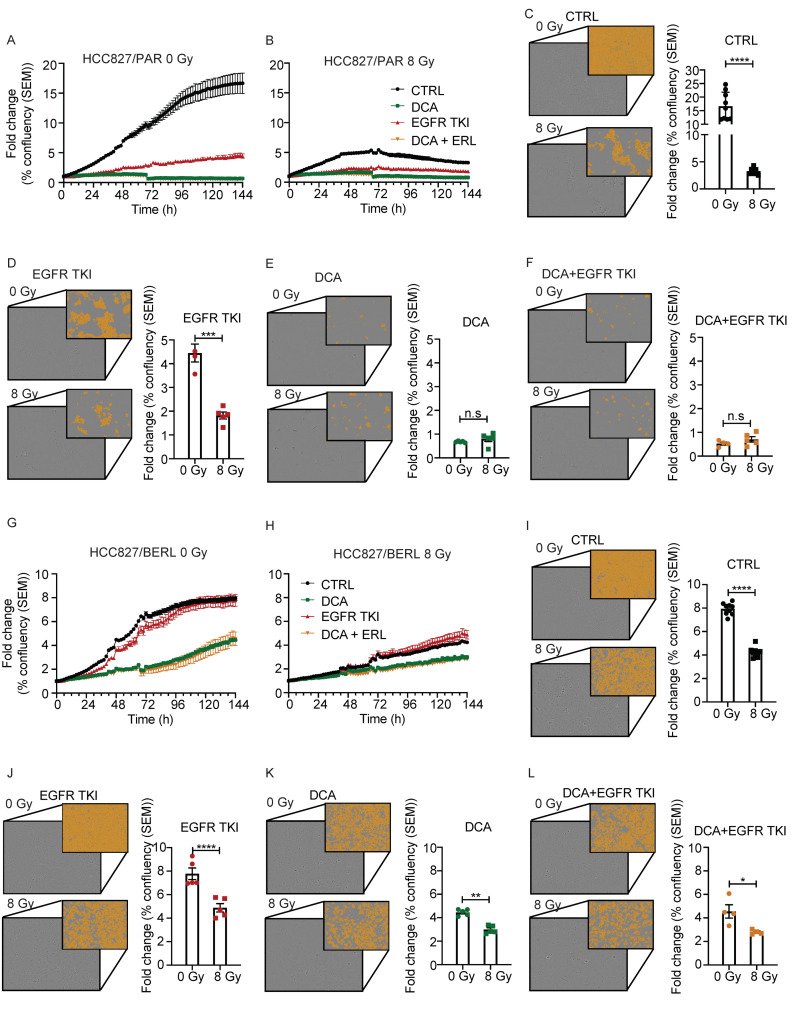
Radiation is increasing the effect of DCA and EGFR TKI in NSCLC cells. Proliferation curves of HCC827/PAR cells treated with 20 mM DCA, 1 µM erlotinib combination of the treatments, without ionizing radiation (**A**) or with ionizing radiation (**B**). Images and column diagram illustrating the effect of ionizing radiation in (**C**) control conditions, (**D**) the presence of EGFR TKI, (**E**) the presence of DCA, or (**F**) in the presence of DCA and EGFR TKI. Proliferation curves of HCC827/BERL cells treated with 20 mM DCA, 1 µM erlotinib combination of the treatments, without ionizing radiation (**G**) or with ionizing radiation (**H**). Images and column diagram illustrating the effect of ionizing radiation in (**I**) control conditions, (**J**) the presence of EGFR TKI, (**K**) the presence of DCA, or (**L**) in the presence of DCA and EGFR TKI. Images and statistics presented are obtained at day 6 after radiation. Both images with contrast as well as images illustrating the masks used for quantitation are included. The presented experiments are representing 1/3 in total, and the technical replicates ranges from 5–10/experiment. Unpaired two-tailed *t*-test were used for statistics (* *p* < 0.05, ** *p* < 0.01, *** *p* < 0.001, **** *p* < 0.0001).

We investigated if the effects of DCA and EGFR TKI could be enhanced in combination with ionizing radiation in both NSCLC cells sensitive and resistant to EGFR TKI. HCC827/PAR and HCC827/BERL cells were treated with ionizing radiation, in clinically relevant doses, 30 min before drugs were added. The proliferation rate was monitored by periodic microscopy. Proliferation curves during 6 days of treatment with EGFR TKI, DCA and the two combined in HCC827/PAR cells is shown in Figure 7A. Figure 7B shows proliferation curves during the same condition in addition to 8 Gy ionizing radiation. The effect of adding 8 Gy is most profound in CTRL cells (Figure 7C), and it also increases the therapeutic response to EGFR TKI compared to only EGFR TKI treatment (Figure 7D). Additive effects of ionizing radiation are however not seen in combination with DCA (Figure 7E), nor with EGFR TKI and DCA in combination (Figure 7F). Proliferation curves during 6 days of treatment with EGFR TKI, DCA and EGFR TKI+DCA in HCC827/BERL cells are shown in Figure 7G. Figure 7H shows proliferation curves during the same condition in addition to 8 Gy ionizing radiation. However, although not as profound as with HCC827/PAR cells, the therapeutic effect of 8 Gy is significant also in HCC827/BERL CTRL cells (Figure 7I). Upon combining 8 Gy with EGFR TKI, the therapeutic response is increased compared to EGFR TKI treatment (Figure 7J). Increased effect of 8 Gy is also seen in combination with DCA, compared to DCA treatment alone (Figure 7K). In addition, the response is significantly increased upon combining EGFR TKI and DCA with 8 Gy compared to 0 Gy (Figure 7L). 

## 4. Discussion

In the presented study, PDHK1 expression was found to be increased in NSCLC tumor tissue compared to normal tissue, and this correlated with increased expression of genes supporting aerobic glycolysis and ROS protection. By using the PDHK antagonist DCA, we redirected pyruvate flux towards mitochondrial oxidation at the expense of lactate production, resulting in antitumor effects for both EGFR TKI sensitive and resistant NSCLC cells. 

As indicated in the presented study, the role of PDHK1, which is a direct target of HIF1α, is correlated to increased glycolysis [36]. HIF1α is a key regulator of hypoxia, a phenomenon related to rapid cancer cell proliferation. In our data, HIF1α expression is correlated to the expression of PDHK1. Based on the link between HIF1α, glycolysis and PDHK1, we hypothesized that targeting PDHK1 would add to the anti-proliferative effects by preventing hypoxia related responses in our cell models. By inhibiting PDHK, the pyruvate pool will be oxidized in the mitochondria, resulting in a reduced activity of the fermentative process where pyruvate is converted into lactate is decreased. 

Transportation of glucose across the cell membrane is dependent on GLUTs. We found GLUT1 to be upregulated in the patient cohorts. We also saw an increase in GLUT2, but not as prominent. Both GLUT1 and GLUT2 overexpression has been linked to poor patient outcome [51,52,53]. To avoid intracellular acidification and apoptosis induced by increased glycolysis, glycolytic cells must sustain both intracellular and microenvironmental lactate homeostasis. In relation to intracellular pH regulation, we found the hypoxia-regulated carbonic anhydrases CAIX and CAXII to be upregulated and correlated to the PDHK-1 on the transcriptional level. Further, the monocarboxylate transporters (MCT) 1–4 and lactic transporters are important to ensure lactate homeostasis, [54,55,56]. According to our studies both MCT1, MCT4 and LDHA are increased in patient tumor samples, compared to normal tissue (Figure 1). Upon lactate fermentation, oxidized NAD^+^ is produced in addition to lactate. NAD^+^ is essential for the conversion of glyceraldehyde-3-phosphate (GAP) to 1,3-bisphosphoglycerate (1,3-BPG) by GAPDH in glycolysis [57], providing another rationale for the cancer cell to keep high fermentative activity. Interestingly, in addition to other genes involved in glycolysis, we also found mRNA of GAPDH, frequently used as a reference gene, to be upregulated in tumor samples compared to normal tissue, indicating that it is not well suited as a reference gene for NSCLC. This finding is in concordance with previously published results [58,59].

ROS is produced as byproducts in the electron transport chain in the mitochondria. EGFR TKIs are known to induce ROS production and mitochondrial dysfunction in NSCLC [15,60]. As shown in numerous of studies, it is thus possible that development of resistance involves increased ROS tolerance [16,17,27,38,39,40,61,62]. In the present study, we also found that many genes involved in the antioxidant defense system were correlated to the expression of PDHK1. These included genes encoding glutathione peroxidases (GPX), as well as thioredoxin- and peroxiredoxin-related genes. Interestingly, and in accordance with our findings, reduced expression of GPX3 has been shown to be linked to increased proliferation rate in NSCLC by modulating redox-mediated signals [63]. 

Supporting the upregulation of many of the genes involved in glycolysis, we also saw a reduction in genes related to gluconeogenesis and mitochondrial biogenesis, with FBP1 and PGC1α being the most deregulated genes. Decreased gluconeogenesis is a phenomenon often related to increased glycolytic activity, and loss of a key gluconeogenesis regulator FBP1 has previously been shown to induce glycolysis, and lead to hyperglycemia and lactic acidosis [64,65,66]. FBP1 downregulation has also been found to reduce ROS production and to be associated with EMT [64,67,68]. In line with downregulated gluconeogenesis, we find genes involved in fatty acid oxidation (FAO) to be decreased in tumor samples compared to NSCLC tissue. One of the genes related to FAO is the PDHK4, another member of the PDHK family. Interestingly, PDHK4 is decreased in the tumor tissue in the patient cohorts included in our study. It is inversely correlated to PDHK1, indicating diverging roles for the two enzymes. We have recently shown that PDHK4 expression is a sensitive marker for FAO [35], however, the role of PDHK4 and the relation to PDHK1 should be further elucidated in future studies. Based on the transcriptional analysis data, we wanted to investigate the potential therapeutic effect by targeting PDHK1 and pyruvate flux in relevant cell models.

The antiproliferative effect is profound upon adding DCA in our cell models. Combining DCA with EGFR TKI increased the effect both in parental as well as in EGFR TKI resistant cells. By rewiring energy metabolism in NSCLC cells, we propose we can restrict cellular plasticity and cancer progression. A previous study has shown that expression of EGFR is associated with increased glucose uptake and glycolysis [69]. Moreover, development of resistance to EGFR TKIs has been shown to cause decreased mitochondrial respiration and increased glycolysis in myeloma cells [15,16,17], indicating that EGFR TKI indeed is affecting the metabolic signature in cells. This finding further justifies the reasoning that inhibition of glycolysis may potentiate the effect of EGFR TKI.

Interestingly, a recently published study shows that DCA also increases the expression of the key tumor suppressor p53 in colorectal cancer, highlighting new possible anticancer mechanisms induced by DCA [70]. Side effects from continuous use of DCA has been few, but include reversible peripheral neuropathy [30]. However, this effect seems to be limited to patients with mitochondrial diseases [71].

The bioenergetic difference between cancer and normal cells may offer selective therapeutic targets. DCA is a pyruvate analogue known to increase lactate and pyruvate oxidation, and to reduce blood glucose levels by inhibition of PDHK [72,73,74]. DCA has primarily been used in patients with lactic acidosis in clinical studies. Wells and colleagues showed that DCA administered in relevant doses in healthy volunteers (15–35 mg/kg/day) reduces lactate and alanine concentrations in an acute manner [75]. As the level of PDHK1 was found to be increased in NSCLC patient tissue, the effects of DCA are encouraging for the design of pre-clinical studies combining DCA or analogs with conventional therapy, including EGFR TKIs. We show that manipulation of pyruvate metabolism using DCA add the effect of EGFR TKIs in NSCLC cell models. DCA was shown to induce a metabolic shift in the cancer cells. In the parental NSCLC cells used in our studies, pyruvate oxidation was increased as expected. However, the overall OCR did not increase substantially, and may suggest that pyruvate flux is not rate limiting for mitochondrial respiration in these cells. However, this needs to be verified in future studies. As reported in other studies [76,77], the acute inhibition of glycolysis was prominent upon DCA addition. This was shown by a significant acute drop in extracellular acidification rate, reflecting reduced lactic fermentation. The rate remained low upon 16 h treatment with 20 mM DCA in culture medium, indicating that the cells are not capable to restore glycolytic activity in the presence of DCA. These finding supports a metabolic shift with enhanced propensity for mitochondrial pyruvate oxidation at the expense of lactate production. Additionally, the transcription levels of lactate dehydrogenase components LDHA and B was decreased upon DCA treatment. In parallel, the mRNA expression of the known marker of mitochondrial biogenesis, PGC1α was increased under the same conditions, supporting the expected response of DCA. 

It is known that an acidic microenvironment is inhibiting the innate immune system in patients [78,79,80]. The deacidification induced by DCA may allow both innate immune system activation as well activation of the adaptive immune system. Ohashi, T. and colleagues has shown that upon DCA treatment, immune cell glycolysis also decreased, improving the immune status, and increasing the effect of anti-tumor immunotherapy [81]. Reducing lactate secretion through inhibition of LDHA has been shown to increase the effect of the immunotherapy including anti PD-L1 through increased activation of inflammatory and antitumor responses [78], and increase T-memory cell activation [79,80]. 

As development of EGFR TKI resistance remains an important contributing factor to the high mortality rate in NSCLC, we created resistant cells to study their metabolic phenotype. These included HCC827 which harbors the EGFR activating deletion mutation E746-A750. H1975 isolated from a primary NSCLC tumor represented the second model. With the additional mutation in the ATP and drug binding domain of EGFR, H1975 cells are resistant to first generation EGFR inhibitors, but remains sensitive to the third generation inhibitor rociletinib. We found that the resistant cells transited from an epithelial to mesenchymal phenotype upon development of resistance, which confirms previous observations [82,83]. EMT is a major trait of carcinoma cell plasticity leading to invasion and metastasis and can also confer acquisition of cancer stem cell properties and development of drug resistance against several cancer therapies [82,83,84]. We did not find that amplification of c-MET contributed to EGFR TKI resistance in our studies (Supplementary Figure S3A).

It appears to be a bidirectional regulatory relationship between mitochondrial energetic function and the EMT program [45,64,85,86]. In line with our previous finding [45], mitochondrial function was reduced upon development of EMT and EGFR TKI resistance. In regard to the DCA induced metabolic shift, we saw the same effect as in the parental cells, however, the HCC827/BERL cells showed an acute increase in the overall OCR in addition. This effect may be due to the fact that the overall OCR is profoundly decreased in this model upon development of EGFR TKI resistance.

In parallel to the presented experiments, we kept cells in culture flasks for more than four weeks to verify that no cells survived and retained proliferation potential upon combining DCA with EGFR TKI’s. These findings indicate that development of resistance towards EGFR TKIs may be counteracted if combined with DCA. Additional in vivo experiments could aid to address the question on development of resistance. When cells have already developed resistance to EGFR TKIs, we experience modest effect of reversing the sensitivity to EGFR TKIs by co-treating with DCA. However, the clonogenic potential is significantly reduced, both when treating with 20 mM DCA alone and in combination with EGFR TKI. Ionizing radiation has high therapeutic effect on the NSCLC cells. For the NSCLC cells sensitive to EGFR TKIs the effect of radiation as monotherapy is more pronounced compared to cells resistant to EGFR TKI. For the EGFR TKI sensitive cells, the additive effect of ionizing radiation in combination with EGFR TKI is clear, compared to cells treated with EGFR TKI (Figure 7D). A reason why we do not see a significant increase of therapeutic potential in these cells upon adding ionizing radiation to DCA or DCA combined with EGFR TKI is likely due to the drastic antiproliferative effect of DCA on these cells. The therapeutic effect of both EGFR TKI and DCA on EGFR TKI resistant cells is significantly increased upon radiation treatment (Figure 7J–L). 

We propose that redirecting the pyruvate metabolism, thus blocking the anaerobic glycolysis, by the use of DCA is creating intracellular stress leaving the cancer cells vulnerable to conventional therapy. As we see a more pronounced response of DCA in parental NSCLC cells, we suggest that the therapeutic timing may be of importance. 

A recent finding has verified that ROS production is increased as a result of DCA [87]. As known, most chemotherapies and ionizing radiation increases cellular stress, in parts by stimulation of ROS production. As we found that ionizing therapy added to the of therapeutic effect of both DCA and EGFR TKI in NSCLC cells resistant to EGFR TKI, also patients with EGFR TKI resistant NSCLC may benefit from addition of both DCA and ionizing radiation. 

## 5. Conclusions

In conclusion, based on our findings in the NSCLC cell models and the two independent patient cohorts, targeting cancer progression through DCA treatment, represents a promising strategy to increase the therapeutic effects initiated by conventional treatment like EGFR TKI and ionizing radiation. DCA treatment facilitates a metabolic shift by inhibition of PDHK activity. This strategy is likely to create metabolic stress that may sensitize NSCLC cells to additional cancer therapy. Based on our findings we propose that targeting PDHK in combination with EGFR TKI and/or ionizing radiation, may increase the therapeutic efficacy and prolong overall survival of patients suffering from NSCLC. Our study supports further exploring PDHK targeting in combination with EGFR TKI, ionizing radiation or other relevant therapies, as promising options targeting the essential mechanisms of cancer progression.

## Data Availability

Data is contained within the article or supplementary material. The results shown in Figure 1, Supplementary Figure S1 and Supplementary Table S1 are in whole or part based upon data generated by the TCGA Research Network: https://www.cancer.gov/tcga (accessed on 5 January 2021).

## Supplementary Materials

The following are available online at https://www.mdpi.com/2072-6694/13/5/941/s1, Figure S1: Transcriptomics data for genes involved in A) Gluconeogenesis and B) fatty acid oxidation and C) Correlation analysis between PDHK1 and PDHK4 expression. D) Expression levels of genes involved in ROS defense. E) Correlation analysis between PDHK1 and genes involved in ROS defense. Figure S2: Resazurin viability assay. Viability of HCC827 (A), H1975 (B) upon increasing concentrations of EGFR TKI and 0 mM, 10 mM, 20 mM and 25 mM DCA. Figure S3: Characterization of resistant HCC827cell model. A) c-MET expression in HCC827 resistant cells compared to parental control. B) Western blot of E-cadherin and vimentin in HCC827 cells. Figure S4: A) E-cadherin shown in Supplementary Figure S3B. B) A-tubulin shown in Figure 4I (upper) and Supplementary Figure S4B. C) Vimentin shown in Supplementary Figure S4B. D) SOD2 shown in Figure 4I. E) Vinculin shown in Supplementary Figure S3B. F) PDHK1 shown in Figure 4I. G) a-tubulin shown in Figure 4I (lower). H) pPDH E1a shown in Figure 4I, Table S1: Statistic values from transcriptomic data.
