# Supplementary material for: Blocking Aerobic Glycolysis by Targeting Pyruvate Dehydrogenase Kinase in Combination with EGFR TKI and Ionizing Radiation Increases Therapeutic Effect in Non-Small Cell Lung Cancer Cells
Source: Cancers (Basel). 2021 Feb 24;13(5):941. doi: 10.3390/cancers13050941 (PMC7956357; doi:10.3390/cancers13050941)
Supplement: Supplementary file 1 [file cancers-13-00941-s001.zip › cancers-1102477-supplementary-final/cancers-1102477-supplementary-final.docx]

Blocking Aerobic Glycolysis by Targeting Pyruvate
Dehydrogenase Kinase in Combination with EGFR TKI and Ionizing Radiation Increases Therapeutic Effect in Non-Small Cell Lung Cancer Cells

Sissel E. Dyrstad, Maria L. Lotsberg, Tuan Zea Tan, Ina K. N. Pettersen, Silje Hjellbrekke, Deusdedit Tusubira, Agnete S. T. Engelsen, Thomas Daubon, Arnaud Mourier, Jean Paul Thiery, Olav Dahl, James B. Lorens, Karl Johan Tronstad and Gro V. Røsland

**Figure S1.** Transcriptomics data for genes involved in **A**) Gluconeogenesis and **B**) fatty acid oxidation and **C**) Correlation analysis between PDHK1 and PDHK4 expression. **D**) Expression levels of genes involved in ROS defence. **E**) Correlation analysis between PDHK1 and genes involved in ROS defence.

**Figure S2.** Resazurin viability assay. Viability of HCC827 (**A**), H1975 (**B**) upon increasing concentrations of EGFR TKI and 0 mM, 10 mM, 20 mM and 25 mM DCA.

**Figure S3.** Characterization of resistant HCC827cell model. **A**) c-MET expression in HCC827 resistant cells compared to parental control. **B**) western blot of E-cadherin and vimentin in HCC827 cells.

**Figure S4. A**) E-cadherin shown in Supplementary Figure S3B. **B**)A-tubulin shown in Figure 4I (upper) and Supplemenary Figure S4B. **C**) Vimentin shown in Supplementary Figure S4B. **D**) SOD2 shown in Figure 4I. **E**) Vinculin shown in Supplementary Figure S3B. **F**) PDHK1 shown in Figure 4I. **G**) a-tubulin shown in Figure 4I (lower). **H**) pPDH E1a shown in Figure 4I.
