# Supplementary material for: Blocking Aerobic Glycolysis by Targeting Pyruvate Dehydrogenase Kinase in Combination with EGFR TKI and Ionizing Radiation Increases Therapeutic Effect in Non-Small Cell Lung Cancer Cells
Source: Cancers (Basel). 2021 Feb 24;13(5):941. doi: 10.3390/cancers13050941 (PMC7956357; doi:10.3390/cancers13050941)
Supplement: Supplementary file 1 [file cancers-13-00941-s001.zip › cancers-1102477-supplementary-final/cancers-1102477-Table S1.pdf]

Tumor vs Normal

Index	Gene	Category	Remark	meanExpr	Normal (n = 110)<br>TCGA LUSC+LUAD (n = 1016) lg2FoldChang	t-test.p_FPKM	TCGA LUSC+LUAD EGFRWT (r lg2FoldChang	t-test.p_FPKM	TCGA LUSC+LUADEGFR MUT lg2FoldChang	t-test.p_FPKM
181	ACOX1	Peroxisomal	PGC1a regula	10,6979667	-0,1254863	0,01313907	0,00288146	0,95153351	-0,0448183	0,33360466
539	ALDOA	Aldolase	---	14,7728858	1,225642	5,91E-68	1,19E+00	4,93E-56	1,08E+00	1,08E-32
1191	ATP1B1	Peroxisomal	---	12,7985035	0,56905391	1,39E-06	7,96E-01	1,17E-10	7,88E-01	2,48E-16
1465	BECN1	Mitophagy, a	---	10,7010373	0,11174195	0,00379063	1,32E-01	1,19E-03	1,90E-01	9,27E-08
1551	BNIP3L	Mitophagy, a	---	11,3963749	-0,4119394	2,11E-11	-3,13E-01	3,83E-07	-3,24E-01	5,00E-06
1552	BNIP3	Mitophagy, a	BCL2 interact	10,421631	1,02910131	2,16E-26	1,12E+00	2,49E-29	8,20E-01	1,06E-19
1597	BRP44L	---	MPC1	8,80534447	-0,6871516	6,50E-20	-5,45E-01	1,44E-13	-3,97E-01	5,72E-06
1598	BRP44	Pyruvate shu	MPC2	9,7523642	0,44783062	3,33E-10	5,37E-01	5,33E-13	5,44E-01	1,07E-10
3004	CAT	Antiooxidant	---	10,7829739	-2,1483842	1,23E-132	-2,00E+00	3,71E-116	-1,85E+00	3,04E-53
4185	CPT2	AMPK target	---	9,35486494	-0,1083167	0,02539014	-0,0225326	0,64290298	0,1701475	0,00230203
4523	CYCS	Mitochondria	---	11,5140808	0,74359105	9,74E-26	6,90E-01	7,06E-20	6,15E-01	2,59E-13
5093	DNM1L	Mitochondria	---	10,520016	0,53007483	8,95E-18	4,28E-01	7,32E-13	3,30E-02	5,31E-01
5569	ENO1	Endolase	---	15,2034136	1,21084953	2,61E-71	1,18E+00	2,25E-62	1,38E+00	2,19E-42
6218	FBP1	Glycolysis rel	know regulat	9,95747423	-2,7582839	5,34E-55	-2,26E+00	3,82E-48	-1,87E+00	3,18E-25
6416	FIS1	Mitochondria	---	10,5746873	-0,2238146	0,00035949	-1,56E-01	1,40E-02	-1,39E-01	1,26E-01
6740	G6PD	Glycolysis rel	---	11,3804709	0,97210525	1,36E-13	8,71E-01	8,44E-11	5,36E-01	2,38E-07
6836	GAPDH	Glyceraldehy	---	15,9315361	2,00446579	4,13E-86	1,86E+00	1,81E-66	1,35E+00	1,95E-32
7076	GLS2	Glycolysis rel	---	6,6917827	0,2178944	0,16216568	0,21326515	0,15701105	0,67543534	0,00012878
7077	GLS	Glycolysis rel	---	11,0712143	-0,6051173	7,51E-09	-5,24E-01	2,02E-06	7,68E-02	4,99E-01
7089	GLUD1	Glycolysis rel	---	11,5161394	-0,1823876	0,0010592	-1,28E-01	1,51E-02	-3,48E-01	1,36E-09
7090	GLUD2	Glycolysis rel	---	8,43317434	-0,2711535	3,94E-05	-2,05E-01	1,96E-03	-3,57E-01	5,31E-08
7373	GPX1	Antiooxidant	Cytosolic, mc	12,1024082	-0,2378236	0,00168171	-0,0837175	0,28290392	0,03094759	0,76641328
7374	GPX2	Antiooxidant	---	8,73911763	4,47863479	5,21E-30	3,96E+00	1,71E-21	2,03E+00	3,31E-08
7375	GPX3	Antiooxidant	HIF target? F	11,4002899	-3,2486804	1,24E-104	-2,97E+00	7,76E-93	-2,77E+00	1,29E-52
7376	GPX4	Antiooxidant	---	11,6888309	0,12054528	0,05959869	0,20602952	0,00227844	0,16517103	0,02113049
7377	GPX5	Antiooxidant	---	0,07700119	-0,0629504	0,07285385	-5,19E-02	1,62E-01	1,03E-04	9,99E-01
7378	GPX6	Antiooxidant	---	0,12551137	0,08172321	0,1189389	0,05652317	0,19673998	0,17114981	0,04527863

7379	GPX7	Antiooxidant	---	7,96373351	0,58911572	4,62E-07	5,16E-01	1,07E-05	9,45E-01	6,36E-15
7380	GPX8	Antiooxidant	---	8,94326036	0,92851496	1,70E-21	9,98E-01	2,10E-22	1,12E+00	2,04E-13
7466	GSR	Antiooxidant	---	10,3147125	0,72221255	1,05E-10	8,02E-01	1,47E-12	2,33E-01	1,46E-02
7776	HIF1A	Glycolysis rel	---	12,2406095	0,68038652	5,18E-18	5,97E-01	1,18E-13	4,25E-01	6,69E-06
7867	HK1	HIF1 targets	metabolic	11,8498008	0,20714727	0,00152275	0,12411562	0,05522452	-0,0581828	0,28855298
7868	HK2	HIF1 targets	metabolic	11,0364675	0,15091747	0,24426764	-0,1752789	0,16472455	-0,213579	0,07187413
9450	LDHA	Glycolysis rel	metabolic	14,0315789	1,32381785	6,98E-66	1,37E+00	2,31E-62	1,13E+00	5,00E-30
9451	LDHB	Glycolysis rel	metabolic	12,9688656	0,57279277	3,09E-08	6,30E-01	2,20E-09	3,88E-01	9,81E-06
9452	LDHC	---	---	2,05287994	0,76354164	0,0002188	0,80327011	0,00019147	1,47008668	3,95E-07
9453	LDHD	---	---	6,8970852	-1,0177287	7,89E-12	-8,98E-01	1,23E-09	-6,63E-01	2,29E-05
10825	MFN1	Mitochondria	---	9,89735071	0,93396853	7,98E-31	7,62E-01	1,02E-21	4,77E-01	1,87E-12
10826	MFN2	Mitochondria	---	11,4343372	-0,1086118	0,03253095	-0,2208892	4,08E-06	-9,56E-02	5,38E-02
11295	MTOR	Glycolysis rel	---	10,4685226	0,28235327	1,01E-07	2,21E-01	2,51E-05	3,91E-01	2,84E-08
11367	MYC	---	---	10,8176237	0,16552793	0,17796225	-0,042383	0,72303518	-0,8399235	3,68E-06
12071	NRF1	Mitochondria	---	8,5807229	-0,10417	0,00019493	-1,39E-01	6,66E-07	-1,78E-01	7,15E-07
12302	OPA1	Mitochondria	---	10,7602652	0,82159832	2,26E-27	6,33E-01	9,11E-19	3,72E-01	1,23E-09
12904	PARK2	Mitophagy, a	---	4,53250703	-1,4234875	1,84E-28	-1,45E+00	3,33E-28	-1,46E+00	8,36E-27
13146	PDK4	Pyruvate shu	metabolic	8,53139341	-3,790971	6,39E-76	-3,24E+00	7,12E-64	-3,30E+00	2,22E-48
13232	PFKFB1	Phosphofruc	---	1,45654942	0,38363307	1,01E-05	2,36E-01	5,78E-03	1,96E-01	9,18E-02
13233	PFKFB2	Phosphofruc	---	9,5994749	-1,0071666	1,79E-40	-9,38E-01	8,81E-32	-7,76E-01	1,12E-13
13234	PFKFB3	Phosphofruc	---	11,265938	-0,6670104	2,66E-13	-7,14E-01	2,52E-13	-5,21E-01	4,79E-07
13235	PFKFB4	Phosphofruc	---	8,04878174	0,72039152	5,73E-12	5,34E-01	5,05E-07	7,49E-01	1,68E-12
13236	PFKL	Phosphofruc	---	11,7940755	0,38120067	9,47E-12	2,85E-01	5,56E-07	4,77E-01	2,89E-12
13237	PFKM	Phosphofruc	---	9,88519246	0,11639749	0,10743749	0,02450702	0,73119494	-0,1328575	0,02542873
13238	PFKP	Phosphofruc	---	11,2124009	1,82279884	1,83E-66	1,81E+00	3,62E-54	1,37E+00	1,66E-22
13410	PINK1	Mitophagy, a	---	10,2988175	-0,7602306	3,80E-31	-6,66E-01	6,22E-27	-3,50E-01	5,34E-07
13459	PKM2	HIF1 targets	metabolic	14,8918503	1,12176503	1,73E-58	1,05E+00	3,46E-47	8,72E-01	5,48E-30
13803	PPARGC1A	Mitochondria	Peroxisome I	5,96055367	-2,3800441	1,67E-28	-2,13E+00	3,71E-22	-2,75E+00	1,30E-33
13804	PPARGC1B	---	---	4,86543255	-0,9195438	5,00E-13	-1,29E+00	2,82E-25	-1,52E+00	3,13E-21
13982	PRDX1	Antiooxidant	We have a p	13,1911965	0,67864303	6,47E-16	6,35E-01	3,28E-14	4,23E-01	2,29E-07
13983	PRDX2	Antiooxidant	---	11,545693	0,66109469	3,83E-16	6,55E-01	1,29E-13	5,30E-01	1,25E-11
13984	PRDX3	Antiooxidant	---	11,3822205	0,30856796	3,59E-07	4,68E-01	6,96E-15	3,98E-01	4,66E-07

13985	PRDX4	Antiooxidant	---	10,9901645	1,22453705	1,47E-54	1,35E+00	2,00E-55	1,25E+00	1,60E-26
13986	PRDX5	Antiooxidant	---	12,1065737	-0,1936927	0,00378933	-0,1481875	0,03134988	0,04970897	0,58420159
13987	PRDX6	Antiooxidant	---	12,3196354	0,18465446	0,00059499	0,22329499	8,48E-05	-0,0216467	0,68154055
14043	PRKG1	Phosphoglyc	---	5,49881232	-2,2215097	1,86E-53	-2,08E+00	6,10E-50	-1,69E+00	5,35E-23
16002	SIRT1	Mitochondria	PGC1a target	8,94856821	-0,46246	4,61E-15	-3,23E-01	2,25E-08	-2,30E-01	2,11E-05
16003	SIRT2	---	---	10,0563202	-0,2180817	0,00015399	-2,87E-01	1,35E-07	-2,58E-01	2,10E-05
16004	SIRT3	Mitochondria	PGC1a target	9,12170297	0,04272485	0,34904011	0,0780822	0,09097159	0,19462635	0,00021381
16005	SIRT4	Mitochondria	PGC1a target	4,19316922	-0,1806836	0,04202238	-0,0034533	0,96888023	0,25238263	0,02934839
16074	SLC16A1	Glycolysis rel	---	9,4356615	1,45227482	3,74E-15	9,42E-01	3,25E-07	3,80E-01	3,87E-02
16075	SLC16A2	---	---	7,97160414	-0,6932121	9,60E-10	-6,36E-01	1,19E-08	-2,50E-01	2,29E-02
16076	SLC16A3	HIF1 targets	metabolic	11,2654915	0,90245398	2,77E-17	1,00E+00	1,67E-19	1,02E+00	2,16E-16
16149	SLC25A1	Mitochondria	---	10,5246807	0,56320212	4,86E-17	4,67E-01	3,76E-12	4,34E-01	2,56E-10
16160	SLC25A2	Mitochondria	---	0,84187482	0,33860916	5,13E-06	2,72E-01	2,09E-04	2,36E-01	1,15E-02
16179	SLC25A4	Mitochondria	Is also regula	9,3855744	-0,7151449	8,66E-23	-5,96E-01	2,84E-16	-4,51E-01	2,09E-09
16211	SLC2A1	Glycolysis rel	GLUT1	12,1747194	4,21028635	9,61E-91	3,70E+00	3,23E-69	3,07E+00	3,89E-39
16212	SLC2A2	Glycolysis rel	GLUT2	0,35629334	0,31831324	0,0034379	0,36217507	0,00160301	0,37022924	0,01856515
16213	SLC2A3	HIF1 targets	GLUT3; meta	10,2408525	-1,5304059	9,20E-31	-1,53E+00	1,02E-30	-1,47E+00	1,93E-19
16214	SLC2A4RG	---	---	10,3837682	0,55507567	5,88E-15	4,65E-01	1,45E-10	5,88E-01	3,35E-14
16215	SLC2A4	Insuline relat	GLUT4; meta	3,9011291	-0,5760155	3,18E-06	-7,33E-01	1,25E-09	-6,36E-01	2,27E-05
16504	SNAI1	HIF1 targets	EMT	6,6574832	-0,735525	2,22E-10	-7,01E-01	7,55E-09	-7,60E-01	1,27E-05
16505	SNAI2	HIF1 targets	EMT	8,4542884	0,83615383	9,64E-07	4,00E-01	1,81E-02	-2,46E-01	1,26E-01
16727	SOD1	Antiooxidant	---	11,6345455	0,50060136	1,06E-16	4,78E-01	1,45E-14	4,51E-01	1,02E-11
16728	SOD2	Antiooxidant	HIF target? F	12,6313716	-0,4867273	6,04E-08	-4,58E-01	4,69E-07	-7,82E-01	1,42E-09
17633	TFAM	Mitochondria	---	9,15601502	0,61817346	1,28E-26	6,68E-01	1,89E-29	5,71E-01	1,54E-16
18218	TOMM20	Mitochondria	---	12,3528954	0,44387403	4,24E-15	4,92E-01	9,08E-17	2,75E-01	3,09E-06
18219	TOMM22	---	---	10,6333921	0,68221583	2,82E-23	6,08E-01	5,76E-17	5,09E-01	2,40E-10
18699	TWIST1	HIF1 targets	EMT	5,33902613	2,56309076	6,81E-34	2,13E+00	7,73E-23	2,04E+00	2,62E-13
18707	TXN2	Antiooxidant	---	10,4894578	0,20041529	0,00013301	1,64E-01	3,07E-03	1,77E-01	6,80E-03
18723	TXNRD1	Antiooxidant	---	12,1928253	0,77004676	1,90E-07	9,09E-01	7,14E-10	-2,71E-02	8,44E-01
18724	TXNRD2	Antiooxidant	---	8,93112951	-0,2574352	0,00012914	-2,27E-01	1,16E-03	-8,03E-02	2,65E-01
18725	TXNRD3IT1	Antiooxidant	TXNRD3	7,73011739	0,21205057	0,00403334	1,58E-01	2,27E-02	1,27E-01	5,36E-02
18726	TXN	Antiooxidant	---	11,8213247	1,00772397	2,80E-22	9,55E-01	4,52E-21	3,61E-01	5,24E-05

18897	ULK1	Mitophagy, a	---	10,4249976	0,71501316	6,68E-26	5,75E-01	1,71E-17	4,46E-01	5,25E-09
18898	ULK2	Mitophagy, a	---	8,79956139	-0,9911451	2,01E-39	-1,01E+00	2,02E-38	-8,52E-01	7,12E-25
19078	VDAC1	Mitochondria	---	11,8308555	0,68284437	6,36E-39	6,77E-01	2,93E-35	4,40E-01	2,92E-12
20106	ZNF746	Mitophagy, a	---	9,09294306	0,35304285	6,91E-10	2,94E-01	3,37E-08	1,93E-01	1,28E-04

			LUAD + LUSC							
**GSE18842**			**TCGA (n = 1016)**		**TCGA EGFRWT (n = 591)**		**TCGA EGFR MUT (n = 76)**		**GSE18842 (*n* = 46)**	
**lg2FoldChang**	**t-test.p_Tumo**	**t-test.paired_**	**vsPDK1_Spea**	**vsPDK1_Spea**	**vsPDK1_Spea**	**vsPDK1_Spea**	**vsPDK1_Spea**	**vsPDK1_Spea**	**vsPDK1_Spea**	**vsPDK1_Spea**
0,13292859	0,10740364	0,067103	-0,2415983	5,49E-15	-0,2465481	1,24E-09	-0,1216405	0,29521254	0,18038853	0,2302731
0,99952952	3,71E-29	2,98E-22	0,32665957	9,68E-27	0,36633231	3,29E-20	0,14731374	0,20411723	0,49972248	0,00040644
0,28988612	0,02202443	0,02548426	-0,2410521	6,35E-15	-0,3154386	4,04E-15	-0,1214491	0,29597868	0,0111625	0,94130759
0,06866077	0,27558311	0,23111381	-0,21561	3,58E-12	-0,2345774	7,86E-09	-0,3326863	0,00332091	0,10292939	0,49606113
-0,330807	0,00044336	0,00047455	0,16007979	2,83E-07	0,17688036	1,52E-05	0,28664388	0,01205919	0,22614863	0,13072262
1,00538966	4,43E-11	1,76E-09	0,33557948	3,24E-28	0,25540355	2,96E-10	0,541162	4,48E-07	0,41325933	0,0043099
0	NaN	NaN	-0,1822192	4,75E-09	-0,0961796	0,01935366	-0,0901435	0,43868983	NaN	NaN
0	NaN	NaN	0,07501148	0,01667677	0,10879919	0,00811555	0,19688312	0,08825602	NaN	NaN
-1,7270265	6,62E-29	1,54E-22	-0,1542073	7,66E-07	-0,1573664	0,00012228	-0,153028	0,18691992	-0,2677151	0,07205759
0,23904652	0,02561921	0,0255096	-0,2849865	1,78E-20	-0,2931566	3,54E-13	-0,2382502	0,03821507	-0,1094665	0,46895122
0,55000032	1,17E-09	4,60E-09	0,34757215	2,81E-30	0,42262848	5,27E-27	0,28820232	0,01158088	0,21381437	0,1536344
0,22455377	0,01308183	0,00779786	0,46240982	4,53E-55	0,51625551	1,44E-41	0,28281613	0,0133074	0,07542399	0,61835516
1,23023389	1,27E-27	1,02E-23	0,27334487	6,67E-19	0,32133218	1,16E-15	0,19887902	0,08501467	0,38328708	0,00855626
-2,8897901	1,66E-17	6,09E-15	-0,4760082	1,08E-58	-0,5278925	1,03E-43	-0,2935612	0,01005975	-0,2735122	0,06587937
-0,338925	2,30E-06	3,92E-06	-0,0412607	0,18837189	0,00523607	0,89892244	0,09971292	0,39143993	0,03139069	0,83593663
0,95879277	1,64E-06	3,78E-06	0,17598481	1,58E-08	0,16095585	8,48E-05	0,19762133	0,08704598	0,53450509	0,00013005
1,28662839	8,54E-37	2,00E-25	0,53939589	6,46E-78	0,56187784	1,76E-50	0,38359535	0,00062499	0,52562442	0,00017603
0,36859104	0,00477783	0,00713852	0,01424392	0,64987719	-0,0130207	0,752091	-0,1100478	0,34396841	0,09750231	0,5191654
-1,3215316	1,26E-09	4,86E-09	-0,1289121	3,70E-05	-0,1087506	0,00814411	-0,1652221	0,15377453	-0,3995683	0,00594051
-0,2008839	0,01230807	0,02815823	0,11885311	0,00014414	0,16613704	4,94E-05	0,21093643	0,06739008	-0,0532223	0,72537351
0,1291644	0,29166423	0,25739619	0,16896767	5,84E-08	0,21686608	1,01E-07	0,29998633	0,0084681	0,08196115	0,58816184
-0,4174354	6,27E-06	1,44E-06	-0,2600967	3,34E-17	-0,1859501	5,35E-06	-0,1062201	0,36111339	-0,3543016	0,01570071
4,29389822	3,27E-16	1,82E-13	0,30255527	5,41E-23	0,24885147	8,59E-10	0,22854409	0,04706149	0,47258711	0,00091208
-2,995349	2,60E-28	5,43E-20	-0,3410086	3,88E-29	-0,3343206	6,72E-17	-0,2642242	0,02108658	-0,3673759	0,01201838
-0,1725053	0,05322575	0,05165679	-0,2244721	4,30E-13	-0,2045739	5,28E-07	-0,1030212	0,37583705	-0,0379278	0,80239419
-0,0610207	0,06049211	0,01650121	-0,0070099	0,82323407	0,03275513	0,4267178	0,02665687	0,81919664	0,14424915	0,33885233
0	NaN	NaN	0,12922884	3,54E-05	0,1699997	3,26E-05	0,0790249	0,49741179	NaN	NaN

0,95726083	1,10E-06	1,57E-06	0,2972095	3,29E-22	0,37286985	6,23E-21	0,24224197	0,0350026	-0,0014184	0,99253545
1,89221409	1,06E-17	7,67E-18	0,07680848	0,01423527	0,21607123	1,13E-07	0,13244019	0,25408475	-0,1384521	0,35882053
1,10565428	3,98E-08	2,18E-07	0,23242258	5,93E-14	0,22709743	2,37E-08	0,3206015	0,00474935	0,38008017	0,00917477
0,5875527	8,87E-09	5,92E-08	0,31006928	4,00E-24	0,35335431	8,04E-19	0,3221326	0,00454242	0,24600678	0,09934909
0,46785777	7,57E-07	4,89E-06	0,17429202	2,18E-08	0,10183217	0,01325698	0,12341763	0,28816057	0,37995683	0,00919931
0,77874405	2,98E-05	5,80E-05	0,47445579	2,85E-58	0,44429748	5,51E-30	0,30673958	0,00703726	0,58741906	1,77E-05
0,82830443	4,90E-22	3,34E-18	0,40552753	1,41E-41	0,46121169	1,82E-32	0,36317157	0,00126221	0,42732038	0,00305692
0,54836963	8,70E-09	2,66E-08	0,13165899	2,51E-05	0,13591481	0,00092452	0,16221463	0,16151067	0,17656491	0,24046924
0,32197194	4,62E-05	1,73E-06	0,06742691	0,03146548	0,12554121	0,00223108	0,28707908	0,01192393	0,2341659	0,11726696
0,01464864	0,87072396	0,96189469	-0,2658477	6,27E-18	-0,2896574	6,90E-13	-0,2661928	0,02011158	-0,1033026	0,49449199
1,36723645	5,66E-20	2,29E-15	0,56849383	3,32E-88	0,6178094	1,72E-63	0,36617908	0,00114134	0,37188936	0,01093206
0,10845217	0,14849664	0,15222617	0,13288908	2,10E-05	0,12088177	0,00324761	-0,1849077	0,10979951	-0,012766	0,93289464
0,296063	1,56E-05	4,36E-06	0,0068989	0,82598854	0,00996138	0,8090442	-0,1303358	0,26177533	0,03817453	0,80113489
0,00187932	0,99278936	0,88477549	0,36579126	1,39E-33	0,34752367	3,22E-18	0,24054682	0,03633808	0,16040703	0,28691783
-0,3202254	8,31E-05	6,76E-05	0,13030294	3,04E-05	0,09573498	0,01992347	-0,0268489	0,81791622	-0,2504471	0,0932026
0,64053878	1,86E-10	1,64E-09	0,4873102	7,86E-62	0,5287563	7,08E-44	0,20880383	0,07027247	0,20185014	0,17855019
-0,1188276	0,02251438	0,02180635	0,05971482	0,0568286	0,03675532	0,37241993	0,05383459	0,64417018	-0,0498319	0,74224656
-3,7017344	8,78E-22	7,49E-19	-0,3741051	3,63E-35	-0,3671966	2,65E-20	-0,2109638	0,06735375	-0,2601912	0,08074919
0,08164684	0,20751359	0,32352693	0,02006769	0,52245887	0,00544557	0,89490106	-0,1665993	0,15032572	0,15127968	0,3155798
-1,1554366	2,21E-12	2,50E-10	-0,2998162	1,37E-22	-0,3256534	4,57E-16	-0,1668353	0,14974056	-0,0452051	0,76546623
-0,5750085	6,47E-05	0,0001193	0,06449097	0,03966026	0,01018728	0,80479997	-0,0020779	0,98578668	-0,0054887	0,97112111
0,5851624	2,69E-05	5,10E-05	0,52077018	7,68E-72	0,49703157	3,37E-38	0,52065619	1,43E-06	0,45593586	0,00145075
0,20098853	0,00265804	0,00047853	0,0337899	0,28144117	0,01763744	0,66872362	-0,1507314	0,19370122	0,14733272	0,32851729
0,55441764	2,60E-06	7,66E-06	0,27951605	9,99E-20	0,32182398	1,04E-15	-0,0158578	0,89185079	0,1684243	0,26318793
1,90920193	1,78E-25	4,37E-19	0,266097	5,83E-18	0,24227702	2,42E-09	0,26343131	0,02149053	0,07542399	0,61835516
-0,6084974	5,51E-11	1,18E-10	-0,4523885	1,67E-52	-0,4748705	1,43E-34	-0,2543814	0,02658839	-0,2931237	0,04804012
0	NaN	NaN	0,36930979	3,00E-34	0,39062675	5,55E-23	0,41626794	0,00018398	NaN	NaN
-1,0036363	4,79E-12	9,30E-10	-0,0792055	0,01147116	-0,0741021	0,07184163	-0,0378401	0,74553606	-0,0308973	0,83848065
-0,7507607	2,12E-07	2,31E-07	0,27065597	1,50E-18	0,28217199	2,79E-12	0,00273411	0,98129897	0,13857539	0,35838838
0,50508323	1,57E-07	4,26E-07	0,27088728	1,40E-18	0,26682928	4,31E-11	0,21006152	0,06856085	0,51945729	0,00021616
0,63817485	1,35E-08	1,69E-09	0,28420735	2,28E-20	0,29973654	9,83E-14	0,1826931	0,11419587	0,42201665	0,00348572
0,17147833	0,00587058	0,00636439	0,05592229	0,07451039	0,16240149	7,31E-05	0,31004785	0,0064174	-0,2684551	0,07124429

0,90503641	4,57E-15	4,14E-14	0,20260903	6,83E-11	0,30656236	2,51E-14	0,44388243	5,91E-05	0,10810977	0,47451185
-0,1974824	0,00961792	0,00772909	-0,1618897	2,07E-07	-0,1210113	0,00321444	-0,1549146	0,18147939	-0,177305	0,23847222
0,32682693	4,54E-07	9,18E-08	0,12409481	7,19E-05	0,09662784	0,01879373	0,18501709	0,10958585	0,29078014	0,04993931
-1,0195696	1,46E-10	3,38E-10	-0,0184572	0,55638222	0,04831584	0,240884	0,00568694	0,96111347	-0,1644773	0,27470162
-0,7077832	8,20E-09	2,16E-07	-0,2627683	1,54E-17	-0,2526864	4,63E-10	0,06515379	0,57603975	-0,3131051	0,03411277
-0,1196523	0,04907561	0,02573049	0,09470488	0,00248836	0,14453152	0,00042394	0,06334928	0,5866755	-0,1238976	0,41201293
-0,0537068	0,22877333	0,13933584	-0,3256579	1,41E-26	-0,3853975	2,30E-22	-0,4143267	0,00019855	0,16250385	0,28058112
-0,0910801	0,06639135	0,03407831	-0,2717221	1,09E-18	-0,2283748	1,97E-08	-0,0136159	0,90706733	-0,2870799	0,05306135
2,37186588	1,84E-15	3,41E-12	0,56116192	1,62E-85	0,59724643	2,07E-58	0,51822283	1,63E-06	0,53623188	0,00012249
-0,1438623	0,32115458	0,22564763	-0,1583529	3,81E-07	-0,0723726	0,0787483	-0,1582228	0,1722183	-0,5045328	0,00034969
0,9735473	2,23E-09	1,89E-09	-0,0822794	0,00862824	-0,122439	0,00286862	0,15029392	0,19501271	-0,0318841	0,83339426
1,0012734	3,67E-14	1,39E-10	0,14841833	1,97E-06	0,07860184	0,0561642	0,12243336	0,29205241	0,25895776	0,0822493
0,04382647	0,19262121	0,26148863	-0,0827353	0,00826501	-0,1405914	0,00060883	0,03674923	0,75263316	0,12229417	0,41813647
-0,6311336	2,03E-09	3,53E-09	-0,1452464	3,27E-06	-0,1538387	0,00017379	-0,1427204	0,21873652	0,11908726	0,43053751
4,27170535	1,08E-30	1,22E-21	0,60166873	2,92E-101	0,6092578	2,48E-61	0,48481203	9,08E-06	0,50724638	0,00032093
-0,0435896	0,09828898	0,08229272	0,00353234	0,91037529	0,02860226	0,48767917	0,03237806	0,78127326	-0,0251002	0,86848836
-1,4088562	5,03E-10	5,11E-09	0,1864598	2,04E-09	0,27588129	8,76E-12	0,20710868	0,07263359	0,06666667	0,65979076
0,32917098	0,00029077	0,00074719	-0,0597259	0,05678243	-0,1220654	0,00295569	-0,1499658	0,1960005	0,06333642	0,67582515
-0,0602525	0,06687427	0,06346485	0,09426461	0,00260708	0,11446917	0,00533508	0,05019822	0,66673242	-0,0623497	0,68060388
-0,461015	0,00017196	0,0001789	0,03989844	0,20339213	0,15737093	0,00012222	0,08997949	0,43952683	-0,3280296	0,02604379
1,16035979	1,37E-06	9,29E-07	0,43723263	8,85E-49	0,44798855	1,63E-30	0,30693096	0,00700002	0,12032069	0,42574368
0,20349131	0,00495953	0,00835364	0,13054535	2,94E-05	0,13938544	0,00067891	0,02252905	0,84682337	0,08985507	0,55260689
-0,6175734	0,00010771	5,40E-06	0,16997399	4,86E-08	0,24754361	1,06E-09	0,33662338	0,00294643	0,04125809	0,78543676
0,63156229	6,36E-09	6,49E-11	0,24840526	8,82E-16	0,34572348	4,92E-18	0,3742447	0,00086713	-0,1156989	0,44386116
0,51092921	1,90E-06	2,81E-06	0,21327148	6,17E-12	0,22246629	4,62E-08	0,29938483	0,00860711	-0,0281838	0,85250127
0,6819483	1,36E-07	1,43E-07	0,43924115	2,91E-49	0,46165118	1,56E-32	0,38996582	0,00049722	0,42843047	0,00297329
1,94860063	1,85E-10	2,99E-10	0,38778178	7,16E-38	0,44302551	8,36E-30	0,2712782	0,0177694	0,1886583	0,20924633
0,23952229	0,00684923	0,00714042	0,19326373	5,05E-10	0,20362445	5,97E-07	0,174108	0,13253058	0,09244527	0,54116658
0,58483426	0,00106735	0,00229358	0,20497765	4,05E-11	0,19413023	1,99E-06	0,39496924	0,00041413	0,37391304	0,01047303
-0,0261809	0,757886	0,62191239	-0,3497148	1,18E-30	-0,4309561	3,99E-28	-0,2128776	0,06484994	-0,1227875	0,41624682
0	NaN	NaN	0,15281415	9,66E-07	0,17811905	1,33E-05	-0,0045933	0,96858741	NaN	NaN
0,48937433	3,72E-05	2,92E-05	0,19345292	4,86E-10	0,16824039	3,94E-05	0,17771702	0,12456825	0,4137527	0,0042593

0,44223704	1,50E-07	2,07E-06	0,13585309	1,37E-05	0,00295599	0,94283309	0,14832536	0,20099336	0,14992291	0,31999012
-0,6657429	1,01E-09	9,17E-09	-0,0012519	0,9681763	-0,0402303	0,32889394	-0,1912782	0,09788192	-0,1036694	0,49295213
0,54490281	5,50E-13	2,18E-11	0,2147433	4,39E-12	0,2640549	6,95E-11	0,17593985	0,12844216	0,21788467	0,14576933
0,04588069	0,5523838	0,55493745	0,14679608	2,56E-06	0,11577999	0,00482949	-0,0021053	0,98559968	0,05704595	0,70649801

r.pv
